# A clinical guide to non-invasive respiratory support in acute respiratory failure: ventilation settings, technical optimization and clinical indications

**DOI:** 10.1186/s13054-025-05730-y

**Published:** 2025-11-18

**Authors:** Emanuele Rezoagli, Alice Nova, Guillaume Carteaux, Marco Giani, Domenico Luca Grieco, Tommaso Pettenuzzo, Alberto Lucchini, Paolo Navalesi, Massimo Antonelli, Giuseppe Foti, Giacomo Bellani, Lise Piquilloud

**Affiliations:** 1 Department of Emergency and Intensive Care, Fondazione IRCCS San Gerardo dei tintori, Monza, Italy; 2 School of Medicine and Surgery, University of Milano-Bicocca, Monza, Italy; 3 Groupe de Recherche Clinique CARMAS, Faculté de Santé, Université Paris Est-Créteil, Créteil Cedex, 94010 France; 4 INSERM U955, Institut Mondor de Recherche Biomédicale, Créteil Cedex, 94010 France; 5 Service de Médecine Intensive Réanimation, CHU Henri Mondor-Albert Chenevier, Assistance Publique- Hôpitaux de Paris, 51, Avenue du Maréchal de Lattre de Tassigny, Créteil Cedex, 94010 France; 6 Department of Emergency, Intensive Care Medicine and Anesthesia, Universitario A. Gemelli IRCCS, Fondazione Policlinico, Rome, Italy; 7 Istituto di Anestesiologia e Rianimazione, Università Cattolica del Sacro Cuore, Rome, Italy; 8 Institute of Anesthesia and Intensive Care, University Hospital of Padua, Padua, Italy; 9 Department of Medicine, University of Padua, Padua, Italy; 10 Centre for Medical Sciences (CISMed), University of Trento, Trento, Italy; 11 Department of Anesthesia and Intensive Care, Santa Chiara Regional Hospital, APSS Trento, Trento, Italy; 12 Adult Intensive Care Unit, Lausanne University Hospital and University of Lausanne, Lausanne, Switzerland

**Keywords:** Non-invasive ventilation, High flow nasal cannula oxygenation, Continuous positive airway pressure, Bilevel positive airway pressure, Facemask, Acute hypoxemic respiratory failure, Acute hypercapnic respiratory failure, Cardiogenic pulmonary edema, Patient self-inflicted lung injury, Asynchronies

## Abstract

Non-invasive respiratory support including high flow nasal therapy (HFNT), continuous positive airway pressure (CPAP) and Bilevel positive airway pressure (BiPAP), exerts distinct physiological effects and requires specific settings and technicalities. HFNT, delivered through dedicated nasal cannulas, provides low levels of positive airway pressure, anatomical dead space washout, allows good patient tolerance and can be used during CPAP or BiPAP breaks. CPAP and BiPAP, administered through various interfaces (e.g., facemasks, oro-nasal masks, or helmets), can deliver higher positive pressure, thereby increasing end-expiratory lung volume, reducing intrapulmonary shunt and oxygenation, with potential benefits on respiratory mechanics as well. BiPAP also delivers pressure support, aiding CO₂ clearance and respiratory muscle unloading, which is especially useful in hypercapnic respiratory failure. Increased intrathoracic pressure also reduces right ventricle preload and left ventricle afterload, which is beneficial in patients with impaired left ventricular function. Non-invasive respiratory support indications depend on the cause of acute respiratory failure. In hypercapnic respiratory failure with respiratory acidosis, BiPAP via facemask is strongly recommended to prevent intubation and reduce mortality. In cardiogenic pulmonary edema, either CPAP or BiPAP is recommended, while HFNT can be useful for patients requiring prolonged support or when CPAP/BiPAP is not tolerated even after ventilator and interface settings optimization. In *de-novo* acute hypoxemic respiratory failure, HFNT should be considered as the first-line treatment, regardless of the aetiology: however, in COVID-19-related AHRF CPAP can be considered to avoid intubation. The choice of non-invasive respiratory support interface in both cardiogenic and non-cardiogenic AHRF should aim at minimizing leaks, optimizing CO_2_ clearance, and maximizing patient tolerance. Monitoring is essential during non-invasive respiratory support to assess patient’s response to treatment and to avoid delaying invasive respiratory support when needed, particularly in hypoxemic patients to avoid intubation delays and prevent patient-self-inflicted lung injury: physiological parameters, clinical scores, and lung ultrasound may help assess the risk of NIV failure. Monitoring tidal volume is valuable but challenging because of leaks. Though not widely used, esophageal pressure monitoring can assess patient effort and transpulmonary pressure. Additionally, electrical impedance tomography is an emerging tool for detecting asynchronous breathing and *pendelluft* phenomena.

**Supplementary Information:**

The online version contains supplementary material available at 10.1186/s13054-025-05730-y.

## Background

Non-invasive respiratory support refers to the delivery of respiratory support with no endotracheal tube or tracheostomy canula in place.

**Fig. 1:**
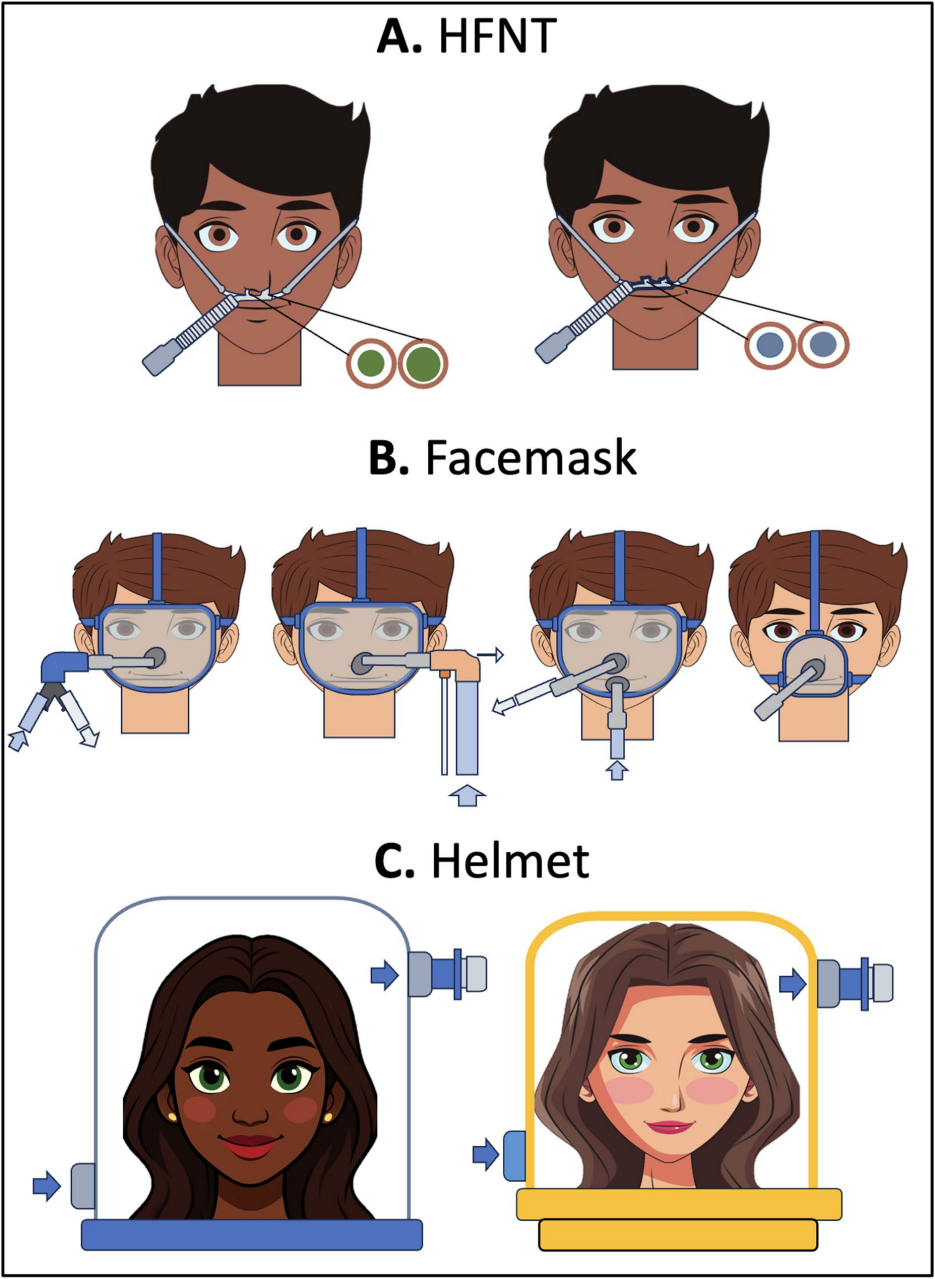
Non-invasive respiratory support strategies and interfaces. **Panel A**. HFNT with asymmetrical (left) and symmetrical cannulas (right). **Panel B**. BiPAP via facemask with double limb circuit vs. single limb circuit and intentional leaks (left); BiPAP via full-face mask with double port vs. BiPAP via oro-nasal mask with one port. **Panel C**. Helmet CPAP vs. Helmet BiPAP. List of abbreviation: BiPAP: bilevel positive airway pressure; CPAP: continuous positive airway pressure; HFNT: high flow nasal therapy

It includes high-flow nasal therapy (HFNT), continuous positive airway pressure (CPAP) and bilevel positive airway pressure (BiPAP). CPAP and BiPAP, often merged under the broad term non-invasive ventilation (NIV), can be applied through various interfaces (Fig. 1) depending on clinical indication, local availability and patient comfort and preference.

Non-invasive respiratory support is widely employed in the acute care setting [1] and clinical indication include acute respiratory failure of various etiologies [2]. During the Coronavirus Disease (COVID)−19 pandemic pandemic [3], the overwhelming number of patients requiring respiratory support, coupled with limited number of ventilators and intensive care unit (ICU) beds, contributed to the further spreading of non-invasive techniques [4, 5].

NIV offer several advantages, including minimal or no sedation requirements, avoidance of muscle paralysis, and reduced risk of ventilator-associated pneumonia. Furthermore, it is feasible and safe outside ICU with proper monitoring [5]. However, NIV drawbacks include patient-ventilator asynchrony, risk of delayed intubation, and, in patients with acute hypoxemic respiratory failure, the risk of patient self-inflicted lung injury (P-SILI).

This review discusses physiological effects and clinical indications of the main non-invasive respiratory support modalities. It focuses on the variety of the commercially available machines and interfaces and the related settings and technical considerations to enhance non-invasive respiratory support performance and patient tolerance.

## Description of non-invasive respiratory support techniques

Non-invasive respiratory support modalities have distinct physiological effects, as well as specific requirements for interface selection, ventilator circuit, technical specifications, and clinical indications. The different non-invasive respiratory support modalities and interfaces are shown in Fig. 1.

## High flow nasal therapy (HFNT)

### From low-flow to high-flow oxygen delivery systems

**Fig. 2:**
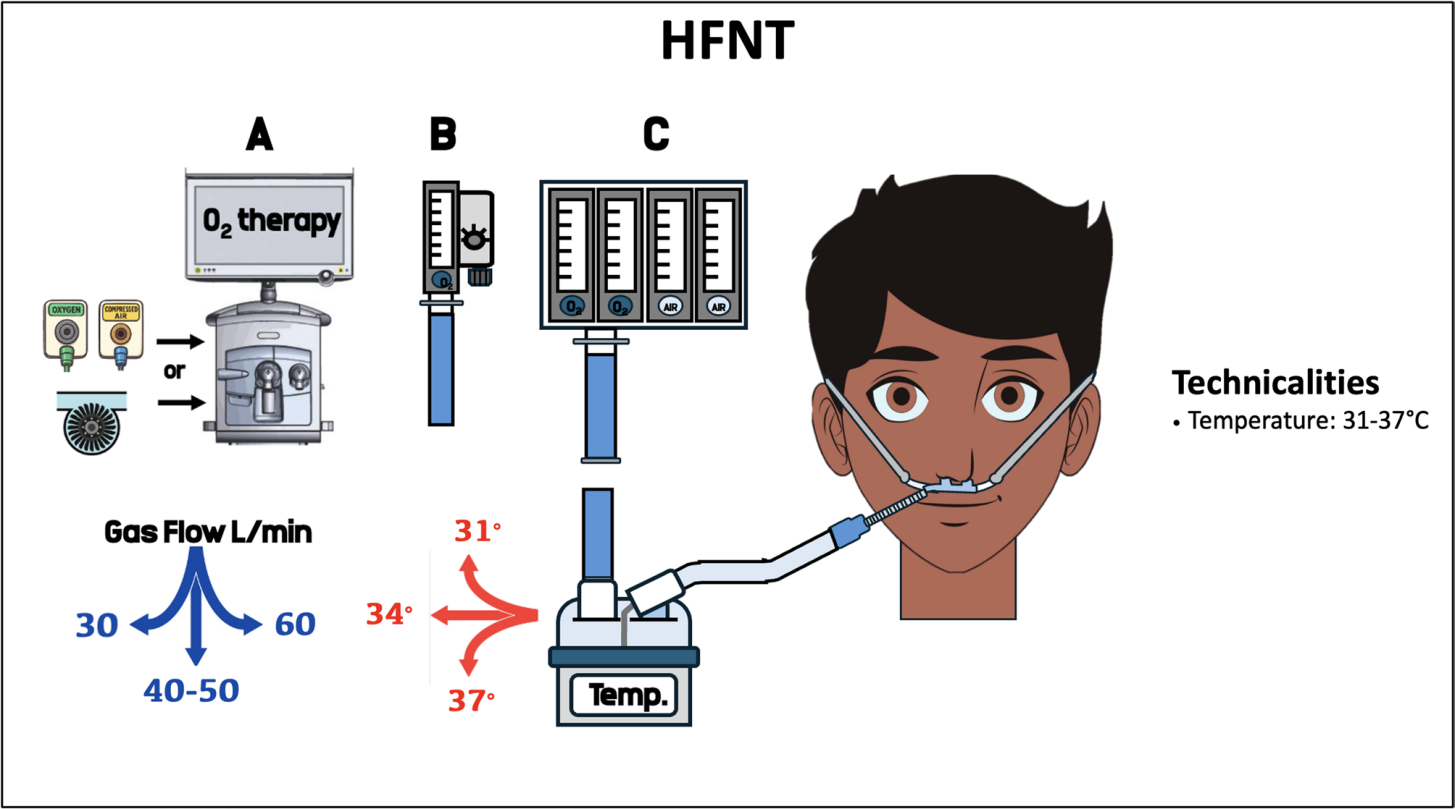
HFNT technicalities. The fresh gas flow can be delivered by a mechanical ventilator (either turbine-driven or pneumatic) set on high-flow O2 therapy (A), Venturi system (B), or oxygen/air blender (C). List of abbreviation: HFNT: high flow nasal therapy

With low-flow oxygen delivery systems, the fraction of inspired oxygen (FiO_2_) can vary significantly depending on the patient’s peak inspiratory flow, the delivery system and the device characteristics [6]. In patients under non-rebreather reservoir bag oxygen mask, it is possible to estimate delivered FiO_2_ by the formula: estimated FiO_2_ = 21%+(flow in L/min*3) [7]. However, this formula does not consider the ambient air entrainment in case of increased inspiratory flow. With flow rates >4 L/min, humidification and warming may not be adequate, limiting patient tolerance and harm airway function [8, 9]. Over the past two decades, devices delivering heated and humidified oxygen at high flows were developed as an alternative to low and intermediate flow (e.g., up to 15 L/min) oxygenation systems. HFNT systems consist of a gas source and a gas mixing system able to generate enough flow (pneumatic or turbine-driven mechanical ventilator, Venturi system or air/oxygen blender) connected to an active heated humidifier with a dedicated circuit. A continuous flow of heated and humidified gas is delivered to the patient through a specific nasal cannula interface [10, 11] (Fig. 2). This facilitates secretion clearance [12], and provide excellent tolerance and comfort, even at the highest flow rate [8].

### Physiologic effects of HFNT

FiO_2_ delivery is flow dependent: the higher the flow, the greater the increase in FiO_2 _[13]. The delivered flow typically matches or exceeds the patient’s inspiratory flow, minimizing room-air entrainment and ensuring a stable delivery of the set FiO₂ throughout treatment [14]. HFNT generates a low level of positive airway pressure (e.g., between 1 and 4 cmH_2_O), depending on the delivered flow rate [15] and whether the patient’s mouth is open or closed [16]. Positive-airway pressure during HFNT increases end-expiratory lung volume in post-cardiac surgery patients with obesity [17] and in non-intubated patients with acute hypoxemic respiratory failure (AHRF) [18], suggesting a role in preventing alveolar collapse [17, 18] and in the homogenizing of gas distribution in the lung [18].

High flow facilitates continuous flushing of carbon dioxide (CO₂) from the upper airways, promoting anatomical dead space clearance [19], which mayy improve alveolar ventilation [20] and reduce minute ventilation and respiratory rate while maintaining similar gas exchanges [21].

Parkle et al. reported mean airway pressure of 1.93 ± 1.25 cmH_2_O, 2.58 ± 1.54 cmH_2_O, and 3.31 ± 1.05 cmH_2_O at flow rate of 30, 40 and 50 L/min, respectively with the subject breathing with mouth closed [15]. Therefore, although HFNT flow rate is usually set between 30 and 60 L/min [11], we suggested a minimum flow of 40 L/min to ensure a minimum PEEP level.

Increasing the flow has been shown to linearly improve respiratory drive, end-expiratory lung volume, and oxygenation in patients with AHRF [22]. A larger cannula diameter, as well as a higher prong area-to-nare ratio, contributes to the generation of higher positive airway pressure, particularly at the upper flow range by reducing leaks around the cannula [23, 24]. However, a larger cannula diameter may cause expiratory [24], prompting mouth opening and pressure loss. Furthermore, the maximal reduction in work of breathing (WOB) might be achieved at a lower flow rate (e.g. 30 L/min) [22]. likely due to more effective CO_2_ clearance from the upper respiratory [25].

For these reasons, clinicians may select the highest tolerated flow, typically starting at 60 L/min, in patients with AHRF to maximize benefits in terms of respiratory mechanics and oxygenation. In contrast, in hypercapnic patients, a lower flow rate (e.g., 30–40 L/min) may be sufficient, as higher flow rate have not been shown to provide additional benefits in reducing inspiratory workload or enhancing CO_2_ clearance [22].

### Asymmetrical HFNT

Recently, asymmetrical prongs were approved as an HFNT interface for clinical use. The asymmetrical cannula features one prong with a smaller diameter and one with a larger diameter, delivering different flow rates to each nostril. The difference in diameter between the two prongs increases the cross-sectional area by 30–40% and results in a greater prong area-to-nare area ratio, which may contribute to higher airway pressure. Additionally, the pressure difference between the nasal cavities, along with the reverse flow pattern from the more occluded to the less occluded and thus less resistant nares, may enhance CO_2_ washout [26]. Asymmetrical HFNT cannulas improve ventilatory efficiency over standard HFNT interface, reducing standardized minute ventilation and WOB in patients with mild-to-moderate hypoxemia [27].

However, despite greater comfort, asymmetrical HFNT showed similar performance in terms of lung aeration, ventilatory efficiency, and inspiratory effort in patients developing acute respiratory failure early after extubation, compared to the standard HFNT interface [28].

### Temperature and humidification

Temperature setting during HFNT may impact patient comfort and tolerance. Higher temperatures offer the advantage of maximal absolute humidity. However, a recent study involving patients with AHRF reported that patient comfort was greater at lower temperature (31°Cvs.37 °C), regardless of the set flow rate [29]. This finding is consistent with data indicating that discomfort can develop due to excessive humidification [30]. Initiating HFNT at a lower temperature and titrating it based on patient comfort may be a reasonable approach to extend the application period and enhance comfort per se.

## Non-invasive ventilation

### Continuous positive airway pressure (CPAP)

#### CPAP delivery systems

During CPAP, a constant positive pressure is applied throughout the entire respiratory cycle. CPAP can be delivered through various types of devices, allowing for adaptation to different environments and constraints [31]. Both helmet and facemask interfaces can be employed. When facemask is used, CPAP can be administered by any kind of ventilator (e.g., ICU, transport, homecare ventilators), either directly in CPAP mode or in pressure support ventilation mode with the pressure support (PS) set to zero. In this situation, delivering pure CPAP is impossible because the ventilator cycles between inspiratory and expiratory phases, resulting in a pressure that is not truly continuous throughout the entire breathing cycle, unlike in pure CPAP mode.

Additionally, CPAP can be provided using easy-to-use, non-electric, single-use devices that require minimal equipment, giving CPAP a frugal dimension and making it suitable for providing respiratory support in resource-limited settings, such as during a pandemic [5, 32–34]. Examples include a fresh gas flow generated by a Venturi system (e.g., Ventumask - Intersurgical, Wokingham, United Kingdom) coupled with a PEEP valve, or virtual valves, that convert gas flow into pressure within the mask (e.g., Boussignac CPAP-Vygon, Ecouen, France- and O-Two CPAP - O-Two medical technologies, Brampton, Canada). Finally, innovative oxygen-saving CPAP have recently been developed (e.g., Bag-CPAP-Air Liquide Medical Systems, Antony, France) [31].

CPAP via low flow systems, such as ventilator CPAP mode, should be avoided, as pressure may not remain constant throughout the respiratory cycle, increasing WOB due to “insufficient flow” (see “Helmet CPAP” section). Therefore, when CPAP is delivered via a mechanical ventilator, using high-flow O_2_ therapy ensures adequate fresh gas flow and stable pressure during the respiratory cycle.

#### Physiologic effects of CPAP

The application of positive end-expiratory pressure (PEEP) increases intrathoracic pressure, influencing both lung function and heart-lung interactions [35]. The primary effect of PEEP on an injured lung is to increase end expiratory lung volume by reopening collapsed alveoli, as long as at least some alveoli can be recruited [36]. This results in a shift of tidal ventilation to the linear portion of the pressure–volume curve, which increases lung compliance, reduces pulmonary shunt, and thus enhances ventilation/perfusion match and improves gas exchange [36]. Additionally, in cases of mild to moderate AHRF not requiring invasive mechanical ventilation, PEEP may help minimize the cyclic opening and closing of atelectatic lung regions (e.g., atelectrauma) [37], stabilizing the alveoli. Although CPAP alone does not increase alveolar ventilation, it can improve CO_2_ clearance and pH, likely by enhancing respiratory mechanics and improved ventilation-perfusion coupling [38].

PEEP has also an impact on both right and left ventricle function. The increase in intrathoracic pressure and right atrial pressure due to PEEP reduces the gradient between mean systemic filling pressure and right atrial pressure, decreasing venous return and, consequently lowering right ventricle preload and stoke volume [39, 40]. This effect of PEEP canreduce interstitial edema, improving alveolocapillary oxygen diffusion [41].

Improved oxygenation, the consequent reduction in hypoxic vasoconstriction, and lower CO_2_ decrease pulmonary vascular resistance [42]. Additionally, alveolar recruitment and increased lung volume further decrease resistance, as long as the PEEP is not too high. Excessive PEEP can compress low-resistance pulmonary capillaries, raising right ventricle afterload, following a U-shaped relationship curve between lung volume and pulmonary vascular resistance [43].

On the left ventricle, CPAP application reduces afterload by minimizing negative swings of intrathoracic pressure associated with spontaneous breathing [44], and by lowering transmural pressure (e.g., the pressure across the left ventricle wall) [45]. These changes enhance left ventricular function and cardiac output, particularly in patients with impaired left ventricle performance and elevated filling pressures. The hemodynamic benefits likely result from reduced preload and transmural pressure, shifting the LV to a more favourable portion on the Frank–Starling curve [46].

In addition, the application of CPAP has been associated with a decrease in airway resistance [47] by reducing the interstitial edema due to fluid accumulation in the peri-bronchial region.

Finally, when PEEP is delivered through a helmet, it acts as a semi-closed “oxygen tent”, ensuring precise FiO_2_ delivery as set by the physician [48]. This may contribute to improved oxygenation alongside lung and heart effects [49]. A ZEEP-PEEP test –evaluating oxygenation and haemodynamics without helmet, after 10 min helmet without PEEP valve and after 10 min with PEEP valve) [50]- helps identify patients who benefit from PEEP and weighs its benefits against risks, particularly when CPAP is poorly tolerated, or potentially harmful (e.g., hemodynamic instability due to hypovolemic status, or high risk of barotrauma).

### Bilevel positive airway pressure (BiPAP)

#### Physiologic effects of bipap

BiPAP ventilation, also known as pressure support non-invasive ventilation (NIV-PS) or non-invasive positive pressure ventilation (NIPPV), is characterized by the application of two levels of pressure: one during the inspiratory phase – delivered as on-demand flow, simultaneously to the patient’s inspiration (e.g.,PS) – and another which is maintained continuously throughout the respiratory cycle (e.g.,PEEP). BiPAP provides similar beneficial effects to CPAP on both lung and cardiac function. Through the application of PEEP, BiPAP increases lung volumes and improves oxygenation [36].

Like CPAP, BiPAP has a positive effect on right ventricle preload and left ventricle afterload, particularly in conditions of heart failure. In addition, BiPAP results in higher minute ventilation, improved CO_2_ clearance, reduced inspiratory effort [51] and WOB [36] compared to CPAP alone [36]. In the presence of “solid-like” injured lung, uneven pleural pressure transmission across the lung parenchyma can cause gas shifts (e.g., *pendelluft*) from the non-dependent to the dependent regions [52, 53], leading to transient overdistension and tidal recruitment in the dependent lung regions during early inspiration. BiPAP may reduce lung stress and *pendelluft* phenomenon by decreasing the patients in inspiratory effort.

However, in some patients with AHRF who do not demonstrate a reduction in inspiratory effort with BiPAP, it may oppositely further increase transpulmonary pressure and tidal volume, exacerbating P-SILI [54, 55].

In chronic obstructive pulmonary disease (COPD), increased small airways resistance, airway collapse, loss of elastic recoil, and increased time constant lead to a predominantly irreversible expiratory airflow limitation. This limitation causes dynamic air trapping in the alveoli at end-expiration, resulting in a higher end-expiratory lung volume compared to the physiological functional residual capacity, and an alveolar pressure higher than atmospheric pressure or set PEEP (e.g., intrinsic PEEP, PEEPi), promoting reabsorption atelectasis, airway closure, impaired gas exchange, and increased WOB [56]. Although in COPD the time constant (product of compliance times resistance) is longer that the time normally required to empty the lungs (e.g., 3 time constants), the presence of irreversible expiratory airflow limitation makes air trapping and PEEPi relatively refractory to change in expiratory time [56].

During acute exacerbation of COPD (AECOPD), inflammation, secretions, and bronchospasm contribute to airflow obstruction and dynamic hyperinflation, leading to increased dead space, impaired gas exchange, hemodynamic compromise, a higher risk of barotrauma, and increased WOB with impaired patient-ventilator interaction due to diaphragm flattening.

Because dynamic hyperinflation is exacerbated by increased respiratory drive, tidal volume, and respiratory rate, BiPAP plays a crucial role in interrupting these pathological mechanisms. The application of extrinsic PEEP in patients with expiratory airflow limitation may reduce PEEPi (e.g.,“PEEP absorber” behavior) by keeping the small airways opened and help maintain a relatively constant end-expiratory lung volume [57]. The addition of extrinsic PEEP also reduces the inspiratory threshold load caused by the difference between airway and alveolar pressure during non-invasive ventilation in presence of dynamic air-trapping, thereby decreasing the WOB.

However, the application of external-PEEP in patients with obstructive pulmonary disease can produce variable effect, thus an external-PEEP trial might be a useful bedside approach [58]. This is easy during invasive controlled mechanical ventilation with muscular paralysis [59], and PEEP is typically set at 80% PEEPi measured at ZEEP [57]. Conversely, during spontaneous breathing this remains challenging. If esophageal pressure monitoring is available, PEEPi can be estimated as the drop in esophageal pressure before the inspiratory flow onset. Gastric pressure monitoring can help distinguishing expiratory muscle activity from hyperinflation [60]. Practical bedside tools for extrinsic PEEP titration include observing accessory muscle activity (e.g., unbalanced PEEPi causes stronger inspiratory effort and accessory muscle contraction) and monitoring patient-ventilator interaction to reduce ineffective efforts and trigger delay [61]. A safe PEEP threshold of 8 cmH_2_O should be applied when BiPAP is applied in obstructive patients. In those with expiratory airflow obstruction (e.g.,asthmatic attack), extrinsic PEEP may combine with PEEPi (e.g.,“PEEP non-absorbers” behavior), worsening air trapping.

Expiratory airflow limitation is also common in morbid obesity, affecting about 20–25% of spontaneously breathing patient [62]. At low lung volume, the small airways tend to collapse, especially when supine, because the abdominal contents limit diaphragmatic movement [63–65], resulting in air-trapping and PEEPi. Additionally, in patients with obstructive sleep apnea, the application of PEEP through NIV can counterbalance PEEPi resulting from small airway collapse, maintain upper airways patency, and reduce the WOB [66, 67].

In BiPAP, patient–ventilator asynchronies can affect patient comfort and treatment success, although it is unclear if they directly worsen clinical outcomes or merely associated with them [68]. The most common types of asynchrony during NIV include auto-triggering and delayed cycling [69], mainly caused by air leaks at the interface.

Expiratory leaks resulting in flow diversion can mimic an inspiratory effort, which can generate auto-triggering [69]. Conversely, in presence of flow-cycled expiration (e.g., expiration occurs at a pre-set percentage of peak inspiratory flow), inspiratory leaks may prevent the flow from reaching the pre-set expiratory trigger [69], resulting in undesired prolongation of the inspiratory time (e.g., delayed cycling). To minimize leaks and better match the patient’s neural expiratory cycling to the ventilator’s set expiratory cycling, it is essential to optimize the interface (e.g., proper size/type of facemask/helmet), adjust ventilator settings (e.g., rise time, PS, PEEP, and expiratory cycling), and use ICU or dedicated NIV ventilators with leaks-compensation software [69]. Compared to conventional flow-cycled mode, a time-cycled expiratory trigger (often indicated as maximal inspiratory time in BIPAP-dedicated ventilator modes) may prevent delayed cycling, reducing respiratory effort and improving patient-ventilator interaction [70].

### Temperature and humidification during NIV

Although the upper airways are not bypassed, humidification during non-invasive respiratory support (CPAP or BiPAP) is recommended, as dry gases ICU ventilators may not be adequately humidified, particularly in mouth-breather or at high inspiratory flow rates, leading to mucosal dryness, reduced tolerance, and worsened bronchial hyperreactivity [71].

Two systems are available for humidification: active humidification via a heated humidifier (HH) and passive humidification via a heat and moisture exchanger (HME). The HME, placed between the Y-piece and the patient, adds dead space to the circuit [72] and slightly increases flow resistance [73]. Its efficacy may be reduced in the presence of significant air leaks. Compared to HME, HH improves alveolar ventilation, CO_2_ elimination [74], and reduces WOB [75]. Although HH has not been shown to affect NIV success rate [76], it is suggested [77]. However, given the strong physiological rationale, HH may be preferred in hypercapnic respiratory failure.

The temperature should be set between 26 and 28 °C, and adjusted according to patient comfort and tolerance. This temperature facilitates an evaporation at least of 10 mg/L of H_2_O vapour [78–80]. For facemask a slightly higher temperature can be set to prevent mucosal dryness and improve patient comfort, as the gas is delivered directly to the airways due to the small internal volume of the mask [81]. In a physiological study in AHRF patients undergoing BiPAP via helmet, a double-tube circuit without external gas conditioning maintained adequate heat and humidity. In this setting, using a HH or HME may cause excessive humidity and heat within the interface, increasing discomfort [30].

HME has the advantages of simplicity and lower cost. Therefore, it may still be used, ideally by optimizing its performance through the selection of filters with low dead space, minimizing additional dead space sources (e.g.,flex tubing), and monitoring for leaks and increased resistance to flow.

## Non-invasive respiratory support interfaces for CPAP and NIV

CPAP and BiPAP can be delivered using either a facemask or a helmet interface.

### Facemask

The facemask is the most commonly used interface for NIV. Facemasks used in critical care can be classified into two main types based on configuration and internal volume:

- *Oro-nasal mask*: covers the mouth and nose, with a lower internal volume (about 100–200 mL) compared to the full-face mask [82];
- *Full-face mask*: covers the whole face and has a large internal volume (approximately 1 L, depending on the manufacturer) [82];

Differences in internal volume across these interfaces do not significantly affect inspiratory effort, breathing patterns, or CO_2_ clearance, suggesting a limited impact on dead space [82–85]. The full-face mask offers advantages in terms of comfort and reduced skin breakdown compared to the oro-nasal mask [83].

**Fig. 3:**
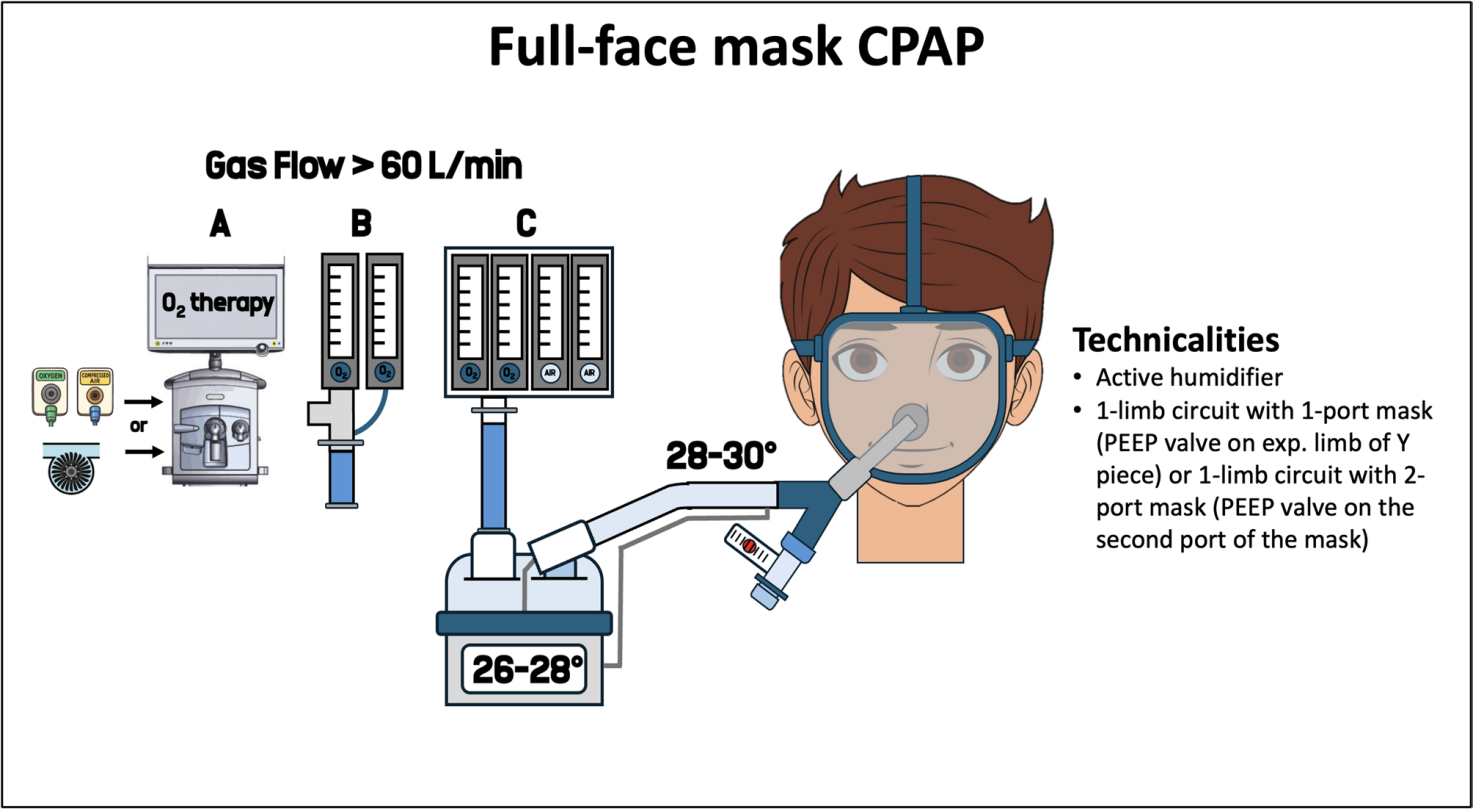
Full-face mask CPAP technicalities. The fresh gas flow can be delivered by a mechanical ventilator (either turbine-driven or pneumatic) set on high flow O_2_ therapy (**A**), Venturi system (**B**) or an oxygen/air blender (**C**). List of abbreviation: CPAP: continuous positive airway pressure; PEEP: positive end-expiratory pressure

**Fig. 4:**
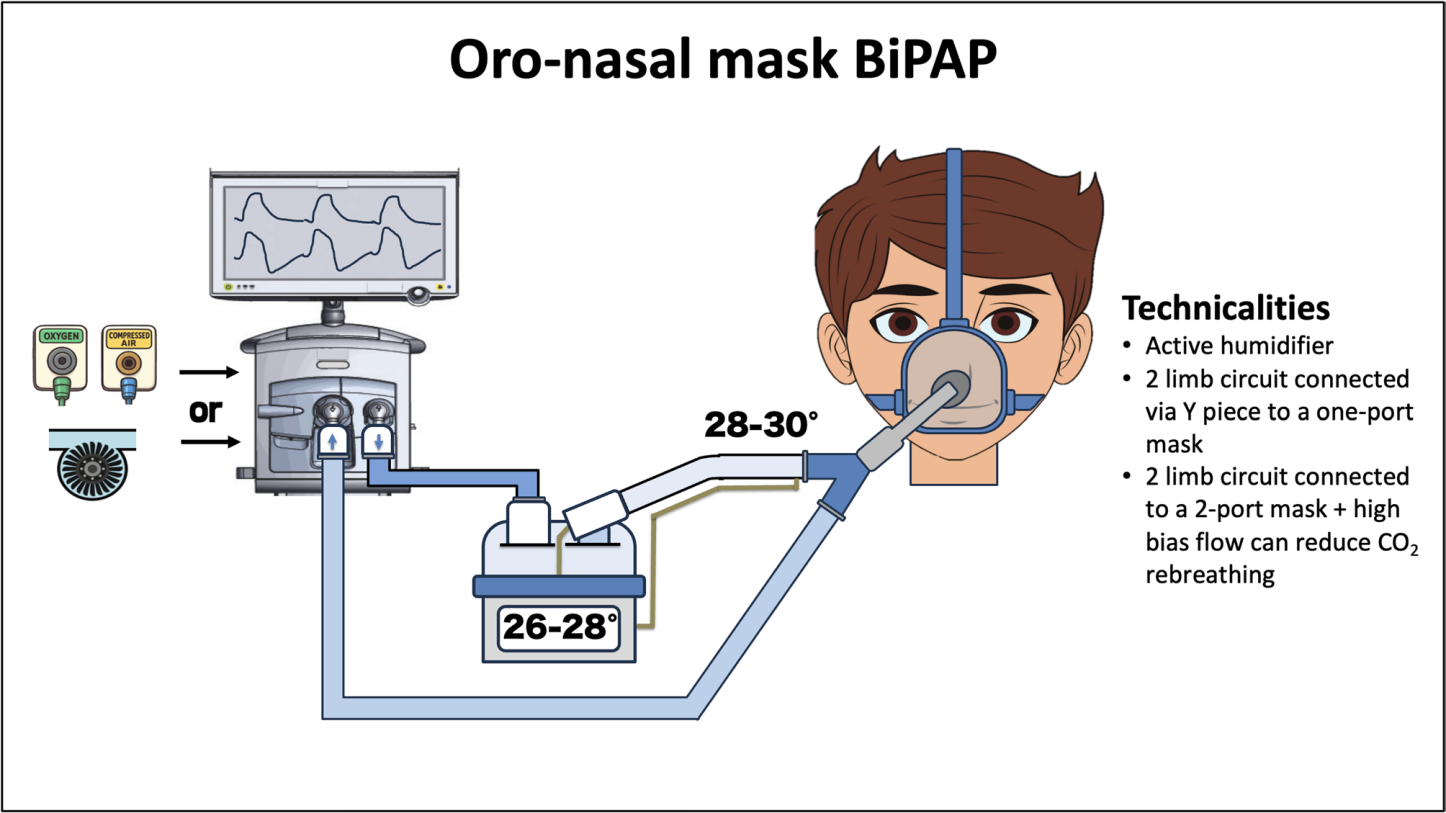
Oro-nasal mask BiPAP technicalities. The BiPAP circuit can be connected to either a turbine-driven or a pneumatic mechanical ventilator. List of abbreviations: BiPAP: bilevel positive airway pressure; CO2: carbon dioxide

The facemask can be connected to either a single-limb or double-limb circuit:

- In a single-limb design, a non-rebreathing expiratory valve allowing exhalation and CO_2_ clearance must be placed in the circuit. Although non-rebreathing expiratory valves allow for full exhalation, they can increase expiratory resistance, potentially impairing patient-ventilator synchrony [86]. Alternatively, when a single-limb circuit is used without a non-rebreathing expiratory valve, an unidirectional exhalation system (e.g., plateau exhalation valve) or intentional leaks (e.g., holes in the mask or in the circuit, whisper swivel) can be used to allow expiration in the ambient air [87, 88]. In healthy volunteers, ventilator circuits without a non-rebreathing valve have been associated with higher minute ventilation and increased WOB, compared to those with a non-rebreathing valve (e.g., “mushroom” or “diaphragm” valve) [86]. When whisper is used, effective CO_2_ removal occurs only with PEEP is ≥ 8 cmH_2_O [89, 90]. In contrast, a plateau exhalation valve ensures complete CO_2_ clearance at any PEEP level [89, 90]. To note, in this configuration, the PEEP valve can be placed either at the Y piece (single-limb circuit with one facemask port) (Fig. 3**)**, or directly on the facemask (single-limb circuit with two facemask ports) [91].

- In double-limb circuits there are separate limbs for inspiratory and expiratory flow either from the Y piece or from the interface. They can have a single-port or a two-port configuration. In the single-port configuration, the most used during NIV, the bias flow does not wash the mask dead space, but it runs along the Y-piece outside the mask (Fig. 4), which limits the effective clearance of dead space, even when high bias flow is applied [91]. In contrast, in a two-port configuration, separate inflow and outflow ports direct fresh gas into the mask’s dead space, reducing CO_2_ rebreathing [91, 92]. In a double-limb circuit with two ports, high bias flow can further minimize CO_2_ rebreathing.

Although commonly used, facemask interface has notable limitations: higher risk of air leaks and asynchronies, inability to eat, drink or communicate without interrupting therapy, and increased risk of skin lesions with prolonged use, reducing tolerance [93]. A recent oro-nasal mask design, applied under the nose to avoid contact with the nasal bridge, may reduce the risk of skin lesions, improve patient comfort, and enhance treatment adherence [94].

### Helmet

The helmet consists of a soft, non-extensible transparent hood secured by a neck-sealing collar without direct facial contact. The collar and anchoring system vary depending on the helmet configuration. The design typically includes two connectors for gas inlet and outlet. Helmets for CPAP (H-CPAP) and BiPAP (H-BiPAP) differ in design and technical specifications.

#### Helmet CPAP (H-CPAP)

**Fig. 5:**
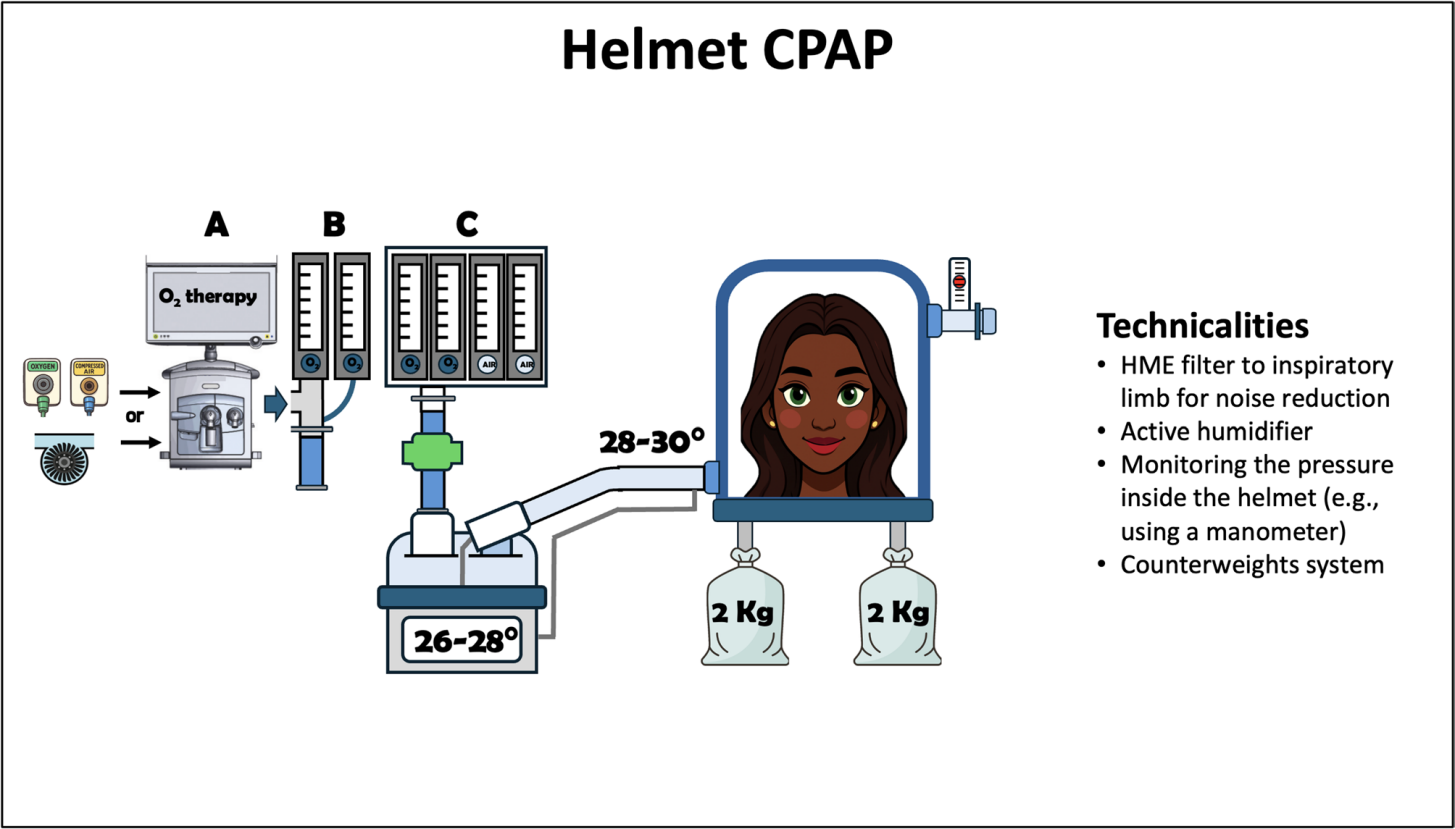
Helmet CPAP technicalities. The fresh gas flow can be delivered by a mechanical ventilator (either turbine-driven or pneumatic) set on high flow O_2_ therapy (**A**), Venturi system (**B**) or an oxygen/air blender (**C**); *the fresh gas flow should be set within the flow range recommended by the manufacturers. List of abbreviation: CPAP: continuous positive airway pressure; HME: heat and moisture exchanger

During H-CPAP, the patient is free to inhale and exhale while the pressure within the helmet remains constant, with no triggered inspiratory support. The configuration of H-CPAP involves a free-flow system and a PEEP valve. A constant fresh gas flow with variable FiO_2_ can be generated by a Venturi system, an oxygen/air blender, or a mechanical ventilator using the high-flow O_2_ therapy option (Fig. 5). A fresh gas flow of at least 60 L/min is essential to maintain the positive pressure level throughout the respiratory cycle by ensuring continuous gas flow through the expiratory valve, and to prevent CO_2_ rebreathing. A fresh gas flow rate below 30–40 L/min can result in significant CO_2_ rebreathing during inspiration [48, 95]. Therefore, CPAP mode on a mechanical ventilator should be avoided for H-CPAP, as its flow would be similar to the patient’s minute ventilation and would not be sufficient for effective CO_2_ clearance [95]. The only safe option to deliver H-CPAP using a mechanical ventilator is the high-flow O_2_ therapy mode, which provides a fresh gas flow of up to 60–80 L/min.

**Fig. 6:**
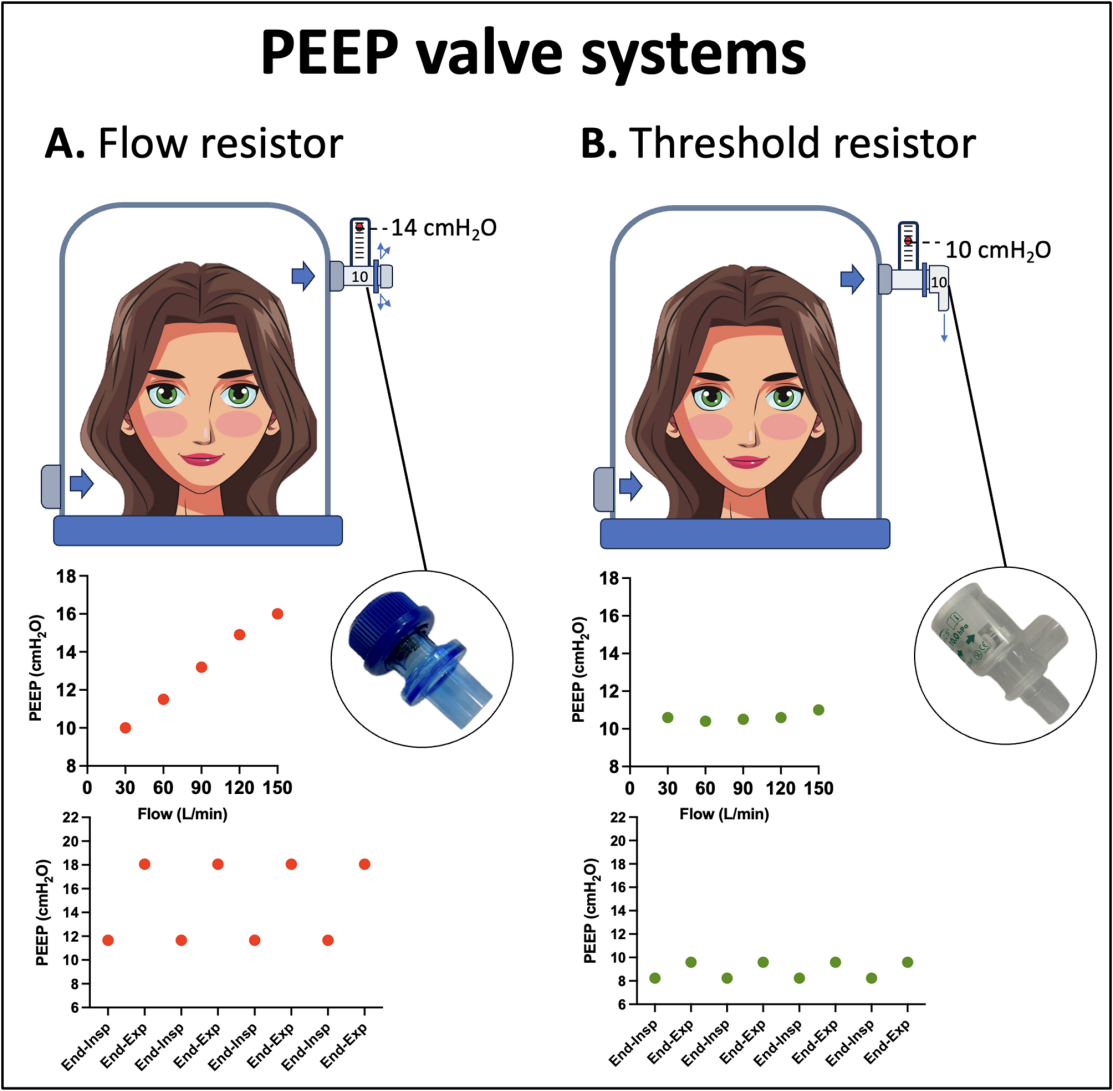
PEEP valve systems. Graphs show representative experimental data obtained under identical setting: PEEP of 10 cmH2O and respiratory rate of 20 breaths per minute. **Panel A**. Adjustable PEEP valve - flow resistor: the PEEP level is flow dependent, resulting in deviation from the target PEEP at different fresh gas flow rates. When the fresh gas flow was set at 90 L/min at the peak inspiratory flow was 68 L/min, large fluctuations of the set PEEP level were observed during the respiratory cycle. **Panel B.** fixed PEEP valve - threshold (Starling) resistor: the target PEEP is generally maintained regardless of the gas flow rate. Under the same condition of fresh gas flow and peak inspiratory flow, the PEEP remained stable throughout the respiratory cycle. List of abbreviations: PEEP: positive expiratory pressure

The gas mixture flows through the helmet and is dispersed via a PEEP valve, which maintains a constant positive pressure in the system [96]. PEEP valve systems can be classified according to their flow-resistive properties into (1) flow resistors and (2) threshold resistors. The flow-resistors - such as a narrow tube or an orifice - generate a flow-dependent pressure, following the equation: pressure = resistance*flow, in the presence of a laminar flow. Therefore, any gas flow variation results in deviations from target PEEP. Conversely, threshold resistors generate pressure by exerting a constant force on a fixed surface area (e.g., pressure = force/surface area), ensuring that target PEEP is maintained regardless of gas flow rate fluctuations (e.g., coughing or change in patient’s effort) (Fig. 6). Commercially PEEP valves - such as water-column, water-seal, adjustable or pre-calibrated spring-loaded valves - act as pure threshold resistors within the manufacturer-recommended flow range [97]. Even threshold PEEP valves may exhibit some degree of flow-dependency at high gas flow rates resulting in pressures above the target PEEP [62]. Additionally, any extra device on the expiratory line (e.g., elbow tube connectors, water traps, HME and HEPA filters) may increase flow resistance, leading to higher pressure than the set PEEP (e.g.,“overpressure”) and increased patient’s inspiratory effort because of higher pressure swings within the helmet [98, 99]. Similarly, a continuous-flow lower than peak inspiratory flow (e.g., “insufficient flow”) can cause large pressure swings within the helmet, resulting in additional and potentially harmful inspiratory effort, particularly in patients with high inspiratory flow demands [99]. A high-compliance reservoir bag [100–102] in the inspiratory line buffers flow variations, minimizes pressure swings, and improves gas humidification by reducing fresh gas flow needs [103].

Therefore, titrating and monitoring the pressure inside the helmet (e.g., using a manometer on the CPAP helmet) [98] is crucial for both preventing complications from excessive pressure [104] and optimizing flow level [97].

During treatment with H-CPAP helmet, certain interventions (e.g., “helmet CPAP bundle”) can enhance patient comfort and device tolerance [105]:

- Avoid armpit braces: A counterweight system should be used instead [105] to prevent pain and pressure ulcers [106].
- Reduce noise: A HME filter on the inspiratory limb significantly reduces airflow noise without affecting pressure delivering [105]. Noise can also be minimized with smooth-bore tubing and earplugs [105].
- Prevent under-humidification: High-flow H-CPAP, particularly using Venturi systems with high FiO₂ or medical gases alone may cause under-humidification [107]. An active humidifier set at 26–28 °C in order to deliver at least 10 mg/L of H_2_O vapour, targeting a temperature around 30 °C within the circuit at the helmet gas inlet (e.g., + 2 °C temperature gradient from the set temperature at the humidifier chamber) [77, 106], when combined with correct gas flow setting, can prevent dryness and condensation. If using an HME filter for noise reduction, the HME should be placed between the medical gas source and the heater chamber inlet [105].

#### Helmet bipap (H-BiPAP)

Although facemask are most commonly used for BiPAP, helmets offer an alternative for non-invasive pressure support. Compared to facemask, the helmet interface allows the application of higher PEEP levels for prolonged time periods, while maintaining the benefits of pressure support in reducing inspiratory effort [51]. This is particularly relevant as Duan et al. found lower NIV failure rate with higher PEEP, primarily due to improved oxygenation [108].When using helmet interface, asynchronies are generally well tolerated and may be not clinically relevant, thanks to the large internal volume of the helmet, which can meet the patient’s flow demands regardless of ventilator response [109]. In addition to its physiological advantages, the helmet al.lows patients to interact with the environment and drink independently through a specific port, minimizing the air leaks and therapy interruption. Furthermore, the helmet reduces the risk of claustrophobia, facilitates early mobilization and physiotherapy, and lowers the incidence of skin ulcers during long-term treatment [93]. However, compared to a face mask, the helmet is noisier and makes managing a jugular central line, placed and secured lower on the patient’s neck, more difficult.

**Fig. 7:**
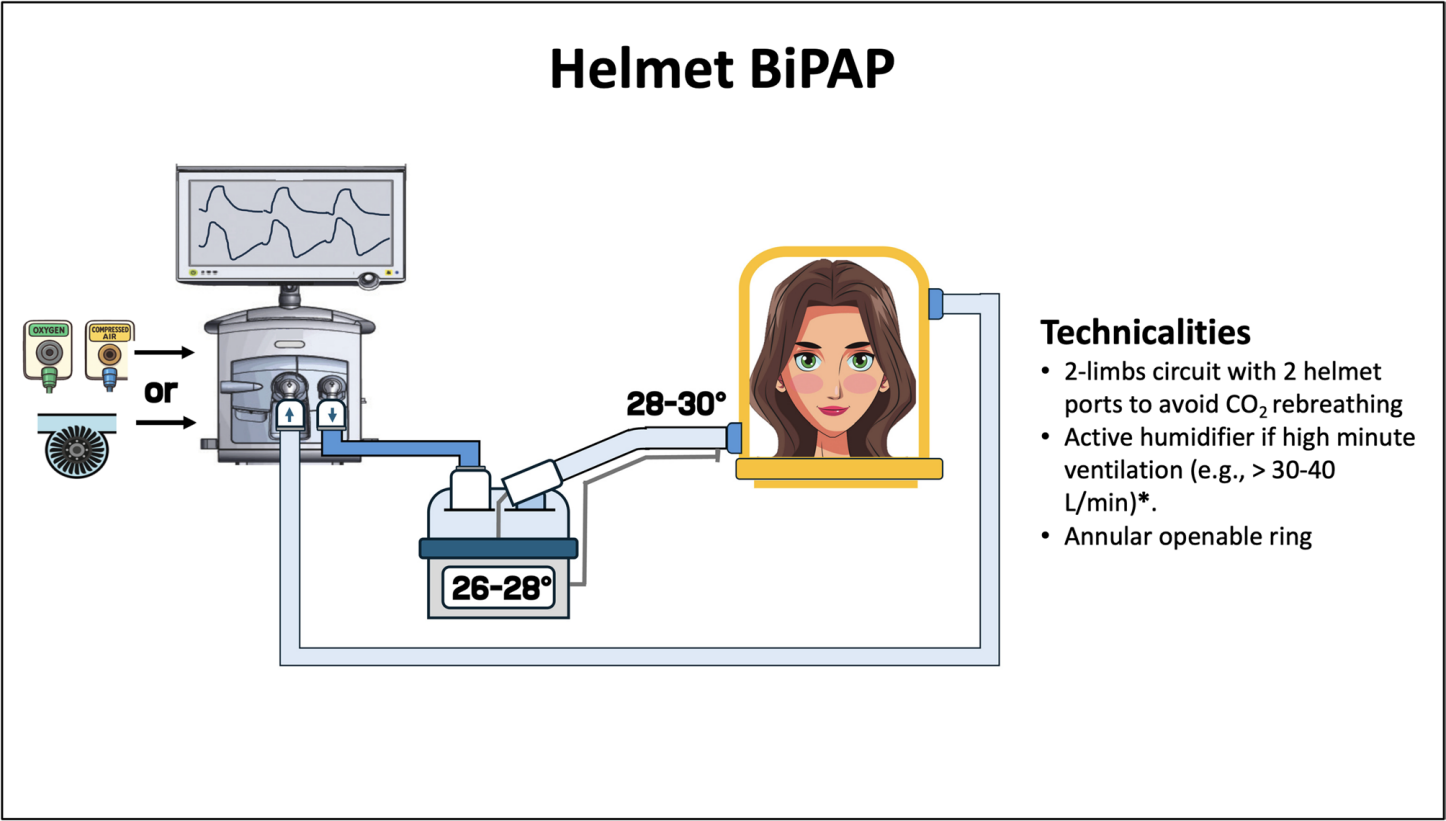
Helmet BiPAP technicalities. The BiPAP circuit can be connected to either a turbine-driven or a pneumatic mechanical ventilator. List of abbreviation: BiPAP: bilevel positive airway pressure; CO2: carbon dioxide. *High minute ventilation refers to the sum of the ventilator bias flow – which is differently represented by the type and brand of the mechanical ventilator - and patient minute ventilation as it is reported on the ventilator screen

Newer helmets specifically designed for BiPAP have smaller internal volume and lower compliance. Additionally, they feature a soft collar, and an opening ring placed beneath the inflatable cushion to prevent displacement during insufflation and reduce the compliance of the patient-interface system, all without armpit braces [110] (Fig. 7). This design improvements result in faster pressurization [111] and enhanced triggering performance, ultimately improving respiratory muscle unloading and patient comfort [110, 112, 113].

If BiPAP is performed using a standard helmet interface (e.g., the same helmet used for CPAP), the large internal volume and the high compliant helmet can lead to delayed pressurization and reduced inspiratory pressure, resulting in patient-ventilator asynchrony [110].

It should be noted that patient-ventilator asynchronies does not always lead to discomfort with helmet BiPAP, as long the patient is able to inhale and exhale in the reservoir of the interface. Additionally, inspiratory de-synchronization may have lung-protective effects, as inspiratory effort and the delivery of pressure support are in part out of phase, limiting the amplitude of transpulmonary pressure swings [114].

To optimize NIV performance by balancing patient-ventilator synchrony, lung protection, respiratory muscle unloading, and CO₂ clearance, specific ventilator settings should be adjusted. These include applying a higher PEEP (e.g.,about 10-12cmH_2_O) to stiffen the helmet, titrating the pressure support level (e.g., about 10-14cmH_2_O) to obtain peak inspiratory flow of 80–100 L/min (HENIVOT2 trial, ClinicalTrials.gov Identifier: NCT05089695), and using a short pressurization time (e.g.,0 Sects.) [11, 114]. However, a recent study reported that H-BiPAP was less effective than facemask BiPAP in reducing dyspnoea in COPD patients with hypercapnic respiratory failure, suggesting that, despite design and setting optimization, a considerable amount of asynchrony remains, likely affecting patient’s perception [115]. CO_2_ rebreathing may be a concern during H-BiPAP, as the helmet’s internal volume is much larger (approximately 18 L) than that of facemask [116]. The average CO_2_ concentration within the helmet depends on CO_2_ production and total helmet ventilation, that can be monitored on the ventilator, unlike patient minute ventilation. Using a two-limb circuit with two independent helmet ports or a single-limb circuit with a modified expiratory valve placed on the helmet’s expiratory port (e.g.,open circuit) improve CO_2_ washout compared to a standard double-limb ventilator circuit connected to the helmet via a Y-piece. Furthermore, a pressure support of 12cmH_2_O typically prevents clinically relevant CO_2_ rebreathing during H-BiPAP ensuring sufficient flow through the helmet.

### Clinical indications

Below, we outline the clinical indications for each non-invasive respiratory support modality based on the cause of respiratory failure.

Table 1 summarize the clinical indications for non-invasive respiratory support in each clinical context, according to the available scientific evidence (Table 1S,2S,3S). Table 2 includes the main non-invasive respiratory support settings for cardiogenic pulmonary edema (CPE), acute hypoxemic respiratory failure (AHRF) and hypercapnic respiratory failure due to acute exacerbation of chronic obstructive disease (AECOPD).

**Table 1.**
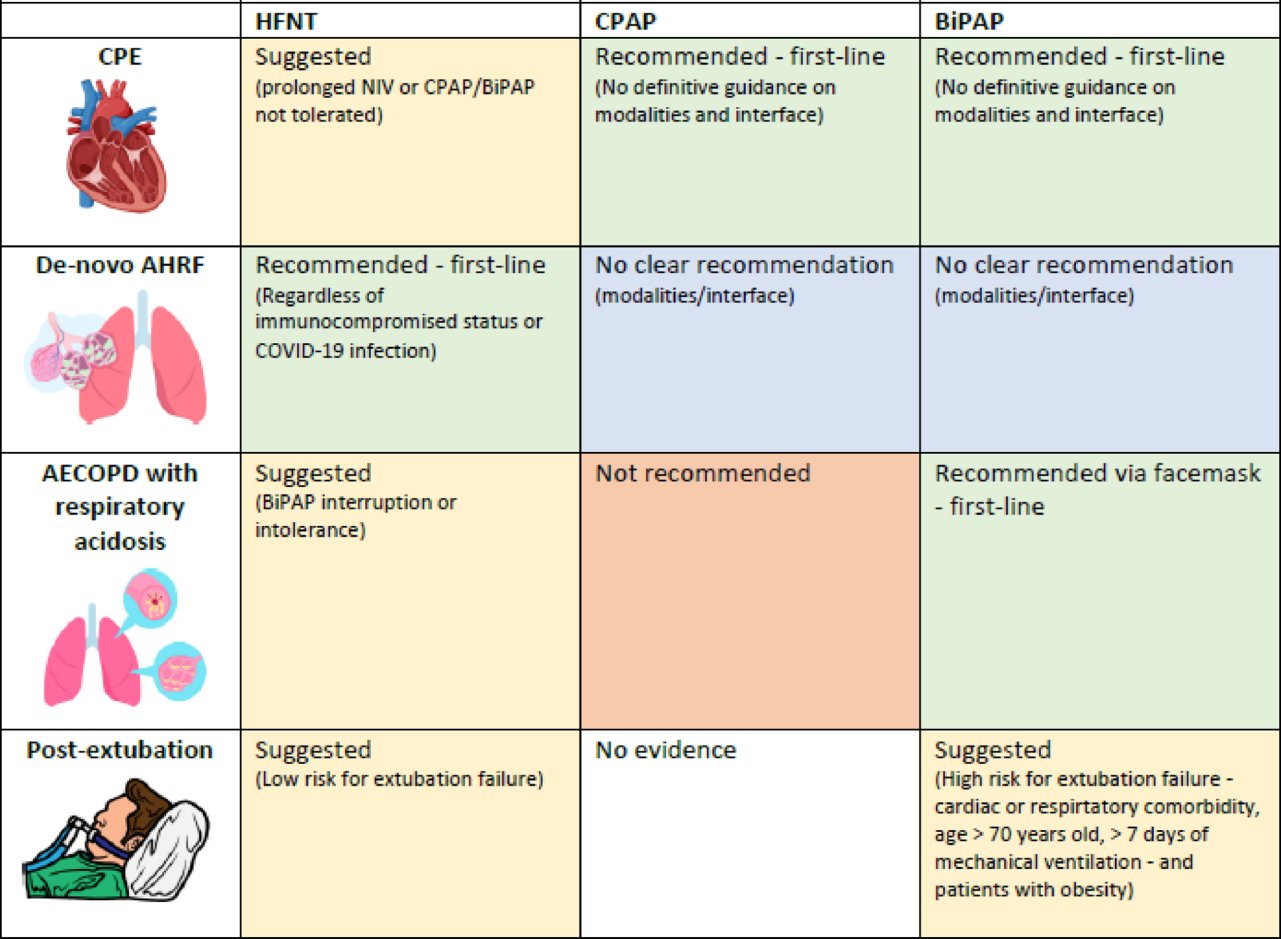
Summary table of clinical indications for non-invasive respiratory support in different clinical settings

AECOPD: acute exacerbation of chronic obstructive pulmonary disease; AHRF: acute hypoxemic respiratory failure; BiPAP: bilevel positive airway pressure; CPAP: continuous positive airway pressure; CPE: cardiogenic pulmonary edema; HFNT: high-flow nasal therapy

**Table 2:** Summary table of main settings for non-invasive respiratory support in different clinical settings

	CPE	De-novo AHRF	AECOPD
**HFNT**	• FiO_2_: 0.21–1 • Gas flow: 40–60 L/min • Temperature: 31–37 °C • Humidification recommended by HH	• FiO_2_: 0.21–1 • Gas flow: 40–60 L/min • Temperature: 31–37 °C • Humidification recommended by HH	• FiO_2_: 0.21–1 (Target SpO_2_ 88–92%) • Gas flow: 30–40 L/min • Temperature: 31–37 °C • Humidification recommended by HH
**CPAP**	• FiO_2_: 0.21–1 • Gas flow: 60–100 L/min (For both helmet/facemask) PEEP: facemask 5–8 cmH_2_O (PEEP may be insufficient to correct hypoxemia; PEEP may be set at higher level for CPAP as leaks have less impact than in BiPAP because there is no synchronization), helmet 8–12 cmH_2_O • Temperature: 26–30 °C (Set 26–28 °C in the humidifier chamber to target ideally 30 °C at the helmet/face mask gas inlet) • Humidification recommended by HH	• FiO_2_: 0.21–1 • Gas flow: 60–100 L/min (For both helmet/facemask) PEEP: facemask 5–8 cmH_2_O (PEEP may be insufficient to correct hypoxemia; PEEP may be set at higher level for CPAP as leaks have less impact than in BiPAP because there is no synchronization), helmet 8–12 cmH_2_O • Temperature: 26–30 °C (Set 26–28 °C in the humidifier chamber to target ideally 30 °C at the helmet/face mask gas inlet) • Humidification recommended by HH	Not recommended
**BiPAP**	• FiO_2_: 0.21–1 PEEP: facemask 5–8 cmH_2_O (PEEP may be insufficient to correct hypoxemia), helmet 8–12 cmH_2_O • PS: facemask 7–10 cmH_2_O, helmet 10–12 cmH_2_O • Temperature: 26–30 °C (Set 26–28 °C in the humidifier chamber to target ideally 30 °C at the helmet/facemask gas inlet) • Humidification: recommended by HH for facemask; not needed for helmet	• FiO_2_: 0.21–1 • PEEP: facemask 5–8 cmH_2_O (PEEP may be insufficient to correct hypoxemia), helmet 8–12 cmH_2_O • PS: facemask 7–10 cmH_2_O, helmet 10–14 cmH_2_O • Temperature: 26–30 °C (Set 26–28 °C in the humidifier chamber to target ideally 30 °C at the helmet/facemask gas inlet) • Humidification: recommended by HH for facemask; not needed for helmet	• FiO_2_: 0.21–1 (Target SpO_2_ 88–92%) • PEEP: facemask 5–8 cmH_2_O • PS: 7–15 cmH_2_O (PS may be increased up to 22 cmH_2_O – keeping and inspiratory positive airway pressure ≤ 30 cmH2O - to target pH 7.35–7.45 by a tidal volume of 10–15 mL/kg of predicted body weight^171^) • Temperature: 26–30 °C (Set 26–28 °C in the humidifier chamber to target ideally 30 °C at the facemask gas inlet) • Humidification: recommended by HH (HH improves alveolar ventilation and CO_2_ clearance)

### Cardiogenic pulmonary edema

In the context of CPE, several randomized controlled trials (RCTs) [117] support the application of NIV (CPAP or BiPAP) to improve clinical outcomes (e.g., reduced need for endotracheal intubation, lower in-hospital mortality). NIV, in addition to pharmacologic interventions, is recommended as first-line therapy for patients with CPE without acute cardiogenic shock or need for revascularization therapy [1]. In CPE, no NIV modality or interface has been shown to be superior [117]; therefore, the choice should prioritize minimizing leaks, ensuring CO_2_ clearance, and optimizing patient tolerance [1, 118]. Even in patients with acidotic CPE, both CPAP and BiPAP reduced hypercapnia, acidosis and heart rate compared to COT [119] (Table S1). Although the first RCT comparing CPAP and BiPAP raised concerns about a potential increased risk of acute myocardial infarction with BiPAP [119], several more recent trials have not confirmed this association [120–123].

In CPE patients, HFNT reduces respiratory rate more than COT [124] and is non-inferior to face mask BiPAP for endotracheal intubation or death within 7 days [125]. However, H-CPAP resulted in a greater short-term respiratory and hemodynamic improvement compared to HFNT [126]. Therefore, HFNT may be beneficial for patients requiring prolonged support or those who do not tolerate CPAP or BiPAP [127].

Due to its simplicity, immediate efficacy, good tolerance and safety, CPAP, delivered via facemask [37, 127], is feasible option in pre-hospital care, where it can be applied by minimally trained personnel. In the context of out-of-hospital CPE, CPAP, delivered via both facemask or helmet, has been shown to provide beneficial effects on respiratory and cardiac function [38, 128, 129].

### De Novo acute hypoxemic respiratory failure

De novo AHRF occurs in patients without chronic respiratory disease, and includes acute respiratory distress syndrome, unilateral pneumonia, and other conditions such as aspiration pneumonia, traumatic lung contusion or consolidation, and septic shock. Non-invasive respiratory support modes can serve as an early alternative to COT, providing positive airway pressure and respiratory muscle unloading while avoiding the adverse effects of intubation.

In the 2015 FLORALI trial, including patients with AHRF and PaO_2_/FiO_2_ ≤ 300 mmHg, HFNT showed similar 28-day intubation rate vs. COT, but more 28-day ventilator-free days, a lower hazard ratio for death at 90 days, and a lower intubation rate in the subgroup of patients with a PaO_2_/FiO_2_ ≤ 200 mmHg [130]. This findings were later confirmed in a small, monocentric trial [131] conducted before the COVID-19 pandemic. HFNT use in AHRF increased during the COVID-19 pandemic, with several RCTs evaluating its advantage over COT in reducing intubation rate [32, 132–135]. A meta-analysis of RCTs and non-RCTs, showed that HFNT was superior to COT in reducing the need for intubation; however, no mortality benefits were observed [136]. In AHRF immunocompromised patients, HFNT did not significantly reduce the intubation rate or 28-day mortality compared to COT [137], and mortality was similar between patients receiving HFNT alone versus NIV alternating with HFNO [138] (Table 2).

When HFNT was compared to BiPAP, the FLORALI trial reported a significantly lower 90-day mortality in the HFNT group [110]. However, subsequent trials found no difference in mortality, and conflicting regarding the intubation rate.

Only a few small RCTs compared CPAP to COT in non-COVID-19 patients, with contradictory results [139–142]. In COVID-19, the RECOVERY-RS RCT reported lower intubation rate and 30-day mortality with CPAP versus COT, while no different between HFNT and COT [32]. CPAP is thus specifically suggested over COT in COVID-19-related AHRF to prevent intubation [143].

BiPAP has been associated with lower intubation rate versus COT [144–150]. A network meta-analysis (25 RCTs, 3804 patients) suggested NIV – whether CPAP or BiPAP – reduces mortality and endotracheal intubation compared to COT [151], although recent studies excluding patients with CPE and chronic lung disease did not confirm this [138, 152, 153].

Therefore, HFNT is currently recommended as the first-line treatment for non-mechanically ventilated patients with non-cardiogenic AHRF in the ICU, regardless of immunocompromised status or COVID-19 infection [143]. Current guidelines from European Respiratory Society/American Thoracic Society and from ESICM do not provide a clear recommendation for or against the use of CPAP or BiPAP instead of COT in the treatment of AHRF [1, 143].

Since RCTs report conflicting results regarding the impact of NIV interface on clinical outcomes [154–156] (Table 2), no recommendation favors helmet over facemask [1, 143]. However, although we suggested lower levels of both PEEP and pressure support with the facemask compared to helmet [11], due to the higher risk of air leaks, and the less compliant structure of the facemask, we acknowledge that, in AHRF, low PEEP level may be insufficient to maintained alveolar recruitment and to correct hypoxemia, thereby exposing the patient to risk of atelectrauma.

### Hypercapnic respiratory failure: acute exacerbation of chronic obstructive pulmonary disease

AECOPD can worsen airway obstruction and hyperinflation, leading to increased alveolar dead space and ultimately resulting in hypercapnic respiratory failure with respiratory acidosis, and increased WOB. BiPAP, can interrupt this vicious circle by unloading the respiratory muscles, reducing the WOB and improving gas exchange.

There is strong evidence supporting the efficacy of BiPAP in preventing endotracheal intubation and reducing mortality compared to COT in patients with AECOPD and respiratory acidosis [157–163]. Two studies [164, 165] have compared BiPAP with invasive ventilation, reporting similar mortality rates between the two treatments, but fewer episodes of ventilator-associated pneumonia, reduced need for tracheostomy, lower risk of hospital re-admission or requirement for long-term oxygen in patients initially treated with BiPAP.

When compared to COT, HFNT has been associated with improved physiological parameters such as WOB, corrected minute ventilation, and PaCO_2 _[166], as well as prolonged time to the next exacerbation [167, 168]. However, in a multicenter randomized trial, HFNT did not reduce the need for invasive respiratory support [169]. Furthermore, RCTs failed to demonstrate any benefit of HFNT over BiPAP in preventing intubation, and a recent non-inferiority trial has failed to demonstrated non-inferiority of HFNT compared to BiPAP in reducing the rate of treatment failure in patients with moderate hypercapnic AECOPD (i.e.,pH 7.25–7.35 and PaCO_2_ ≥ 50 mmHg) [170]. Conversely, the RENOVATE RCT demonstrated non-inferiority of HFNT compared with facemask BiPAP for endotracheal intubation or death within 7 days [125]. A recent RCT showed that high-intensity BiPAP reduced the need for intubation in AECOPD patients with respiratory acidosis compared to low-intensity BiPAP, with crossover patients also improving pH and PaCO₂, supporting higher pressure use to avoid invasive ventilation [171] (Table S3).

Given its established efficacy in reducing mortality and intubation rates, BiPAP via facemask is the first-line therapy for acute-on-chronic respiratory acidosis due to AECOPD [1, 172]. A trial of BiPAP should also be considered for patients with severe hypercapnic acidosis even in presence of altered consciousness before mechanical ventilation [173], unless they are rapidly deteriorating [1, 172]. In cases where BiPAP is not tolerated or during BiPAP cycles interruptions, HFNT may be considered as an alternative [172, 174].

### Prophylactic non-invasive respiratory support after extubation

In ICU, non-invasive respiratory support is often applied immediately after extubation to preserve the benefits of mechanical ventilation, such as lung recruitment and respiratory muscle unloading, while reducing the risk of respiratory failure and reintubation. In patients at high risk of extubation failure (e.g., mechanical ventilation for more than 24 h, age >65 years, presence of comorbidities) BiPAP is recognized as an efficient strategy to reduce the risk of reintubation [1].

In this population, Hernández et al. reported that HFNT was non-inferior to BiPAP in preventing reintubation and post-extubation respiratory failure [175]. However, more recent trials showed a lower reintubation rate when BiPAP was used instead of HFNT [176], or when it was combined with HFNT [177], compared to HFNT alone (Table S4). These evidences were further confirmed by recent meta-analysis [178, 179]. In patients with obesity, the application of positive pressure after extubation helps maintain end-expiratory lung volume and prevent upper airway collapse. A post-hoc analysis of a multicenter RCT [177] suggests prophylactic BiPAP alternating with HFNT may decrease the risk of reintubation and mortality versus HFNT alone [180].

In low-risk patients without the previously described risk factors for extubation failure, HFNT has been compared with COT, but findings have been inconsistent. [166, 181–183].

Current guidelines suggest NIV over HFNT for patients at high risk of extubation failure after ≥ 24 h of mechanical ventilation [174]; in lower-risk patients, HFNT is suggested over COT, although the evidence level is low [174]. NIV should be applied for prolonged periods within the first 24–48 h following extubation (i.e.,for at least 12 h within the first 24 h) [176, 177, 184], and tolerance should be optimized through careful interface selection, device rotation, active humidification, and appropriate NIV setting.

### Other areas of clinical implementation of NIV: acute decompensation of obesity hypoventilation syndrome (OHS)

OHS is a severe form of obesity-induced respiratory compromise, defined by the coexistance of obesity, sleep-disordered breathing, and daytime hypercapnia (awake resting PaCO_2_ ≥ 45 mmHg) [185]. The pathophysiology of OHS involves reduced functional residual capacity due to obesity, impaired central response to hypercapnia and hypoxia, sleep-related breathing disorders, and neurohormonal abnormalities. In stableambulatory OHS, positive airway pressure therapy improves the control of the obstructive sleep apnoea, enhances sleep quality, and optimize daytime gas-exchange [186–188]. Therefore, CPAP is suggested as first-line treatment for outpatients with OHS and concurrent severe obstructive sleep apnea [185]. OHS exacerbation is a common cause of acute-on-chronic hypercapnic respiratory failure, often requiring ICU admission, particularly in the presence of respiratory acidosis. Compared to normocapnic patients with obesity, those with OHS have higher ICU admission rates and mortality [189]. However, no formal guidelines for non-invasive management of the acute OHS decompensation, as most studies focus on ambulatory chronic patients. In the acute setting, BiPAP is considered the therapy of choice, as it maintains upper airway patency, unloads respiratory muscle, and improves alveolar ventilation [190]. Full face mask are more effective than nasal mask in acutely ill patients, as they allow the delivery of higher pressures with reduced leakage [191].

### Other areas of clinical implementation of NIV: pulmonary exacerbation of cystic fibrosis (CF)

CF lung disease is characterized by progressive airflow obstruction due to mucus plugging, bronchial inflammation, and destruction of the lung parenchyma secondary to bronchiectasies, progressively leading to increased respiratory muscle workload to ensure adequate ventilation [192]. The clinical course of CF is characterized by “pulmonary exacerbations”, due to the inability of the respiratory muscles to meet the increased ventilation demand (e.g., respiratory tract infections), leading to hypercapnic respiratory failure. During acute exacerbations of CF, BiPAP is frequently used to unload the respiratory muscles, increase alveolar ventilation and enhance gas exchange [192]. Although no RCTs are available comparing NIV to invasive ventilation, observational studies have shown that BiPAP is a reasonable first-line treatment option for hospitalised patients with severe respiratory exacerbations of CF, given the poor outcome of the patients undergoing invasive ventilation [193, 194]. HFNT therapy may serve as a viable alternative or complement to BiPAP, as it has demonstrated physiological benefits, such as reducing respiratory rate and minute ventilation [195].

#### Monitoring the patient with non-invasive respiratory support

Non-invasive strategies are particularly effective in CPE and AECOPD with respiratory acidosis. In CPE, CPAP and BiPAP serve as hemodynamic treatments in addition to providing respiratory support [36, 38, 41]. Consequently, once non-invasive support is initiated in case of CPE, signs of respiratory distress and blood gases typically improve rapidly. In hypercapnic patients (e.g., AECOPD), BiPAP has been associated with a intubation rates reductionfrom 75% to 10–15%^159^. In these patients, NIV failure is linked to persistent acidosis or hypercapnia rather than hypoxia [159]; In such a situation, if the interface and ventilator settings have already been optimized, intubation must be considered.

During *de novo* AHRF, non-invasive respiratory support is rather a double-edged sword. Indeed, as in assisted invasive mechanical ventilation, the negative pleural pressure generated during inspiration can be harmful and may exacerbate existing lung injury (e.g., P-SILI) through several mechanisms [196]. Primarily, the negative pressure leading to elevated transpulmonary pressure, results in global or regional excessive pressure/volume changes [197]. Additionally, negative pleural pressure deflections increase vascular transmural pressure and, in presence of inflammation and epithelial damage, vascular permeability, contributing to alveolar flooding [198]. If insufficient PEEP is applied, cyclic alveolar opening and closing may lead to atelectrauma [37], while PEEP can lead to overdistention, negatively affecting pulmonary vascular resistance. Finally, the inhomogeneous transmission of pleural pressure in the presence of “solid-like” injured lung can cause *pendelluft* [52], potentially overstretching the dependent lung regions [53, 199]. As spontaneous breathing may be harmful and deterioration rapid, close monitoring during non-invasive respiratory support in AHRF is essential, using physiological parameters, clinical scores and advanced tools to identify injurious effort and to predict NIV failure.

Moderate-to-severe hypoxia (e.g., PaO_2_/FiO_2_ < 150 mmHg) has been associated with a higher risk of NIV failure and worse outcomes, including increased ICU mortality [2], raising concerns about the use of NIV in patients with severe AHRF. The following factors have also been associated with facemask NIV failure during de novo AHRF: age greater than 40 years [200], extra-respiratory organ failure [2, 200–202], persistent hypoxemia [202–204], an increase in PaCO_2_ during the first 48 h of treatment [2], and worsening radiologic infiltrates 24 h after admission [205]. High tidal volume has been independently associated with NIV failure and 90 days mortality [203], with volumes greater than 9 mL/kg has been identified as a strong predictor of intubation [203, 206]. During BiPAP, injurious tidal volumes and transpulmonary driving pressures may result from both excessive inspiratory effort or PS. Moreover, when patient inspiratory effort remains relatively constant (i.e., does not decrease in response to PS), tidal volume and transpulmonary driving pressure increase as a function of PS level and lung compliance [207]. Consequently, the absence of decrease or even the increase in inspiratory effort, coupled with excessive PS, may result in non-protective ventilation, which can cause NIV failure [54], and further exacerbate lung injury [55]. These pathophysiological mechanisms may explain some concerning findings associating BiPAP to increased risk of mortality. Retrospective data from COVID-19 patients indicated that BiPAP was associated with the highest mortality rate compared to CPAP and HFNT, even after adjustment of major covariates [208]. Furthermore, a recent analysis of the Extracorporeal Life Support Organization Registry [163] found prolonged BiPAP use before intubation - versus other non-invasive supports or the absence of NIV - was independently associated with higher hospital mortality in severe COVID-19 patients requiring extracorporeal membrane oxygenation.

However, monitoring tidal volume in non-intubated patients remains challenging, especially with HFNT, helmet interface or single-limb NIV circuits [139, 209]. Tidal volume cannot be estimated using a low-level CPAP test during HFNT [210], and it is not reliably measured with helmet interface.

In contrast, it can be quantified by the mechanical ventilator during BiPAP via facemask and double limb circuit. Bio-impedance-based monitoring devices may help detect harmful breathing patters [211–213], but clinical data on its application remain limited. Despite its relatively invasive nature and the need for additional equipment and expertise, monitoring esophageal pressure swings may assist clinicians in decision-making for patients undergoing NIV, particularly in assessing the need for endotracheal intubation. A **≥** 10 cmH_2_O drop in esophageal pressure swing within 2 h of NIV has been shown to be a strong predictor of treatment success [54], while esophageal pressure swing during BiPAP correlates with radiographic progression, suggesting P-SILI as a potential mechanism of lung injury worsening in these patients [54].

A post hoc analysis of the HENIVOT trial found that helmet BiPAP improved outcomes over HFNT in patients with PaCO_2_ < 35 mmHg, whereas no benefit was seen in normocapnic patients [214], suggesting that hypocapnia may be a warning sign of injurious inspiratory effortrequiring escalation of respiratory support. Clinical scores have also been developed to predict NIV failure. The HACOR score – including Heart rate, Acidosis, level of Consciousness, Oxygenation (PaO_2_/FIO_2_), and Respiratory rate - can be easily measured at the bedside. A HACOR score >5 after 1–2 h of NIV has demonstrated good predictive performance for NIV failure in both AHRF [215] and hypercapnic respiratory failure [216]. The respiratory rate-oxygenation (ROX) index is a simple tool to identify patients treated with HFNT who are at high risk of deterioration [217]. The ROX index has also been validated for predicting COVID-19-related CPAP failure [218]. Furthermore, integrating tidal volume into the Volume-OXygenation (VOX) index (SpO_2_/FiO_2_ to tidal volume) has shown to be a robust early predictor of HFNT failure in patients with AHRF [219]. Ultrasonography is a widely used bedside tool for monitoring the clinical course of respiratory failure in critical care setting. In COVID-19-related respiratory failure, lung ultrasound (LUS) has been shown to be a reliable predictor of clinical outcomes, including endotracheal intubation and mortality [220]. The LUS score, alone [220] or combined into the Lung Ultrasound Score (LUSS)/ROX index [221], may help clinicians identify patients who could benefit from NIV and prevent delays in initiating invasive support. Ultrasound assessment of diaphragm thickness and thickening fraction provides insights into respiratory muscle function [222]. Diaphragm thickening fraction and respiratory rate/diaphragm thickening fraction ratio may serve as simple, non-invasive tools to predict NIV outcomes [223].

End-tidal CO_2_ (P_ET_CO_2_), measured via a mainstream sensor between patient’s nose and mouth under the facemask, correlates well with PaCO_2_ values and, may aid early recognition of respiratory failure [224].

Finally, electrical impedance tomography (EIT) may provide valuable insights for the safe management of spontaneously breathing patients. In a pilot study evaluating COVID-19 patients undergoing CPAP, a reduction in end-expiratory lung impedance following PEEP de-escalation was shown to predict poor recruitment and CPAP failure [225]. Although computing absolute tidal volume is not always practical or feasible, EIT allows accurate monitoring of respiratory rate [226] and regional ventilation. By integrating ventilation distribution data with flow and/or airway pressure signals, it help assess patient-ventilator interaction and detect asynchronies [227]. Additionally, EIT can assist in identifying *pendelluft* through various methods [52, 228].

The main monitoring parameters predicting failure or success of non-invasive respiratory support, along with their established cut-off values, are summarized in Table 3

**Table 3:** Main monitoring parameters and tools with corresponding cut-off values for patients with AHRF undergoing NIV

Monitoring tool	NIV modality	Cut - off
PaO_2_/FiO_2 _[2]	CPAP, BiPAP	< 150 mmHg (Cut-off to predict NIV failure)
Tidal volume [203, 206]	HFNT, BiPAP	> 9 mL/kg (1 h after treatment) (Cut-off to predict NIV failure)
ΔPes [54]	BiPAP	ΔPes reduction ***≥*** 10 cmH_2_O (2 h after treatment) (Cut-off to predict NIV success)
HACOR score [215]	BiPAP	> 5 (1 h after tratment) (Cut-off to predict NIV failure)
ROX indez [217, 218]	HFNT, CPAP	< 3.85 for HFNT, < 6.32 for CPAP (6 h after treatment start) (Cut-off to predict NIV failure)
VOX index [219]	HFNT	< 22.67 (6 h after treatment start) (Cut-off to predict NIV success)
LUSS [220]	HFNT, CPAP	> 33 (Cut-off to predict NIV failure)
LUSS/ROX index [221]	HFNT, CPAP	> 4.64 (Cut-off to predict NIV failure)
Diaphragm thickening fraction [223]	BiPAP	< 36.3–37.1% (Cut-off to predict NIV failure)
Respiratory rate/Diaphragm thickening fraction [223]	BiPAP	> 0.6 (Cut-off to predict NIV failure)

AHRF: acute hypoxemic respiratory failure; BiPAP: bilevel positive airway pressure; CPAP: continuous positive airway pressure; ΔPes: esophageal pressure swing (negative deflections of Pes from the onset of inspiratory effort); HACOR: Heart rate, Acidosis, level of Consciousness, Oxygenation, Respiratory rate; HFNT: high-flow nasal therapy; LUSS: Lung Ultrasound Score; ROX: respiratory rate-oxygenation; VOX: Volume-Oxygenation

## Conclusions

Non-invasive respiratory support is widely used for acute respiratory failure in and outside ICUs.

HFNT requires a specific interface and offer several benefits, including low-level positive airway pressure, anatomical dead space clearance, and humidification, ensuring comfort and tolerance. Both CPAP and BiPAP increase end-expiratory lung volume, reduce intrapulmonary shunt, and improve respiratory mechanics. In patients with CPE, they support cardiac function by lowering LV afterload and RV preload. BiPAP effectively improves minute ventilation and CO_2_ clearance while unloading respiratory muscles and decreasing WOB.

CPAP and BiPAP can be delivered through either a facemask or a helmet. With facemask, separate inflow and outflow ports, combined with a high bias flow, help reduce CO₂ rebreathing, while during H-CPAP, a fresh gas flow rate above 30–40 L/min prevents CO₂ rebreathing. Both “overpressure” (from high gas flow and/or additional devices) and “insufficient flow” (from strong inspiratory effort) should be avoided. Helmets with low internal volume and compliance should be used for H-BiPAP to reduce the risk of asynchronies.

In CPE, either CPAP or BiPAP should be used, while HFNT benefits patients requiring prolonged NIV or intolerant CPAP/BiPAP. In de-novo AHRF not requiring immediate intubation, HFNT should be considered the first-line treatment. There is no clear recommendation for using CPAP or BiPAP over COT; however, CPAP/BiPAP is preferred in COVID-19-related AHRF to prevent intubation. The choice of NIV interface in both cardiogenic and non-cardiogenic AHRF should aim to minimize leaks, ensure effective CO_2_ clearance, and maximize tolerance. In case of respiratory acidosis, BiPAP via facemask is strongly recommended since it prevents endotracheal intubation and reduces mortality.

Close monitoringwith physiological, clinical, and bedside toolsduring NIV is essential to prevent P-SILI and avoid delayed intubation. 

## Supplementary Information

Supplementary Material 1

## Data Availability

No datasets were generated or analysed during the current study.

## Abbreviations

AECOPD: Acute exacerbation of chronic obstructive pulmonary disease
BiPAP: Bilevel positive airway pressure
CPAP: Continuous positive airway pressure
CO_2_: Carbon dioxide
COPD: Chronic obstructive pulmonary disease
COT: Conventional oxygen therapy
EIT: Electrical impedance tomography
FiO_2_: Fraction of inspired oxygen
HACOR: Heart rate, Acidosis, level of Consciousness, Oxygenation, Respiratory rate
H-CPAP: Helmet continuous positive airway pressure
HEPA: High efficiency particulate air
HME: Heat and moisture exchanger
HFNT: High flow nasal therapy
H-BiPAP: Helmet bilevel positive airway pressure
ICU: Intensive care unit
NIV: Non-invasive ventilation
PEEP: Positive end-expiratory pressure
P-SILI: Patient self-inflicted lung injury
PS: Pressure support
LUS: Lung ultrasound
RCT: Randomized controlled trial
ROX: Respiratory rate-oxygenation
VOX: Volume-oxygenation
WOB: Work of breathing
