# Supplementary material for: A clinical guide to non-invasive respiratory support in acute respiratory failure: ventilation settings, technical optimization and clinical indications
Source: Crit Care. 2025 Nov 18;29:496. doi: 10.1186/s13054-025-05730-y (PMC12625427; doi:10.1186/s13054-025-05730-y)
Supplement: Supplementary file 1 — Supplementary Material 1 [file 13054_2025_5730_MOESM1_ESM.docx]

**SUPPLEMENTARY MATHERIAL**

**A CLINICAL GUIDE TO NON-INVASIVE RESPIRATORY SUPPORT IN ACUTE RESPIRATORY FAILURE: VENTILATION SETTINGS, TECHNICAL OPTIMIZATION AND CLINICAL INDICATIONS.**

Emanuele Rezoagli^1,2^, Alice Nova^1^, Guillame Carteaux^3,4,5^, Marco Giani^1,2^, Domenico Luca Grieco^6,7^, Tommaso Pettenuzzo^8,9^, Alberto Lucchini^1,2^, Paolo Navalesi^8,9^, Massimo Antonelli^6,7^, Giuseppe Foti^1,2^, Giacomo Bellani^10,11^, Lise Piquilloud^12^

**Table 1S.** Main RCTs comparing different non-invasive respiratory support techniques in CPE (chronological order of publication)

| **Author, year, N centers** | **N Patients** | **Techniques** | **Interface** | **Outcomes** |
| --- | --- | --- | --- | --- |
| Mehta, 1997^1^, 1 ED | N=27, CPE | CPAP (N=13) vs. BiPAP (N=14) | Nasal mask | *Clinical improvement at 30 mins:* significant RR decrease with CPAP vs. significant pH, RR, HR, PaCO2, and BP improvement with BiPAP; *Incidence of AMI:* CPAP 31% vs. BiPAP 71% (p=0.06). |
| Bellone, 2004^2^, 1 ED | N=46, CPE | CPAP (N=22) vs. BiPAP (N=24) | Facemask | *Incidence of AMI*: CPAP 13.6% vs. BiPAP 8.3% (p>0.05); *EI:* CPAP 4.5% vs. BiPAP 8.3% (p>0.05). |
| Crane, 2004^3^, 1 ED | N=60, CPE with acidosis (pH<7.35) | COT (N=20) vs. CPAP (N=20) vs. BiPAP (N=20) | Facemask | *Treatment success:* COT 15% vs. CPAP 35% vs. BiPAP 45% (p=0.116); *Hospital survival:* COT 70% vs. CPAP 100% vs. BiPAP 75% (p=0.029). |
| Park^4^, 2004, 1 ED | N=80, CPE | COT (N=26) vs. CPAP (N=27) vs. BiPAP (N=27) | Facemask | *EI rate at 24 hours:* COT 42% vs. CPAP 7% vs. BiPAP 7% (p=0.001); *hospital mortality*: COT 23% vs. CPAP 4% vs. BiPAP 7% (p=0.061). |
| Bellone, 2005^5^, 1 ED | N=36, CPE | CPAP (N=18) vs. BiPAP (N=18) | Facemask | *Resolution time*: CPAP 29±18 vs. BiPAP 30±16 (p=0.87); *EI*: CPAP 5.5% vs. BiPAP 11.1% (p=0.50); *in-hospital mortality*: CPAP 5.5% vs. BiPAP 0% (p=0.50). |
| Ferrari, 2007^6^, 1 ED | N=52, CPE | CPAP (N=27) vs. BiPAP (N=25) | Facemask | *Incidence of AMI:* CPAP 26.9% vs. BiPAP 16% (p=0.244); *EI:* CPAP 0% vs. BiPAP 4% (p=0.481); *in-hospital death:* CPAP 7.4% vs. BiPAP 12% (p=0.662). |
| Moritz, 2007^7^, 3 EDs | N=109, CPE | CPAP (N=59) vs. BiPAP (N=50) | Facemask | *EI, death, or AMI (combined):* CPAP 5% vs. BiPAP 12% (OR 0.4, CI 95% 0.0-1.9). |
| Gray, 2008^8^, 26 EDs | N=1156, CPE with acidosis (pH<7.35) | COT (N=367) vs. CPAP (N=346) vs. BiPAP (N=356) | Facemask | *7-days mortality:* COT 9.8% vs. NIV (CPAP or BiPAP) 9.5% (p=0.87); *mortality or EI (combined) within 7 days*: CPAP 11.7% vs. BiPAP 11.1% (p=0.81). |
| Ferrari, 2010^9^, 3 EDs | N=80, CPE | CPAP (N=40) vs. BiPAP (N=40) | Facemask | *EI:* CPAP 0% vs. 7.5% (p=0.241); *in-hospital mortality:* CPAP 5% vs. BiPAP 17.5% (p=0.154). |
| Liesching, 2014^10^, 1 ED | N=27, CPE | CPAP (N=14) vs. BiPAP (N=13) | Oronasal mask | *Incidence of AMI:* CPAP 0% vs. BiPAP 7.7% (p=0.97); *ICU admission:* CPAP 92% vs. BiPAP 38% (p<0.05); hospital *mortality*: CPAP 14.3 % vs. BiPAP 7.7% (p=0.084); *clinical improvement*: lower dyspnoea score and higher PaO_2_/FiO_2_ after 30 minutes in BiPAP vs. CPAP (p<0.05). |
| Osman, 2021^11^, 1 ED | N=188, CPE | HFNT (N=94) vs. CPAP (N=94) | Helmet | *Mean RR change at 1h:* HFNT -9 vs CPAP -12 (p<0.001); *Mean HR change at 1h:* HFNT -15 vs CPAP -20 (p=0.042); *Mean PaO2/FiO_2_ change at 1h:* HFNT 120 vs CPAP 149 (p=0.003); *median HACOR score change:* HFNT 4[-2; 9] vs CPAP 6[0; -12] (p<0.001); *median dyspnea scale*: HFNT -3.5[-1; -6] vs CPAP -4 [-1;-7] (p 0.003). |
| Marjanovic^12^, 2024, 3 EDs | N=60, CPE | HFNT (N=30) vs. BiPAP (N=30) | Unspecified | *Median RR change at 60 min:* HFNT −10 [−12; −8] breaths/min vs. BiPAP −7 [−11; −5] breaths/min (p=0.052); *median SpO_2_ change at 60 min*: HFNT 2 [0; 5] vs. BiPAP 2 [-1; 5] (p=0.60); *dyspnea level and patient discomfort*: no difference between the 2 groups. |
| RENOVATE Investigators and the BRICNet Authors^13^, 2024, 33 centers (EDs, medical wards or ICUs) | N=272, CPE subgroup | HFNT (N=136) vs. BiPAP (N=136) | Facemask | *Death or intubation (combined) within 7 days:* HFNT 10.3% vs. BiPAP 21.3% (OR 0.97, 95% CI 0.73-1.2; noninferiority posterior probability 0.997). |

List of abbreviations: AMI: acute myocardial infarction; BiPAP: bilevel positive airway pressure; BP: blood pressure; CPAP: continuous positive airway pressure; COT: Conventional oxygen therapy; CPE: cardiogenic pulmonary edema; ED: emergency departments; EI: endotracheal intubation; FiO_2_: inspired fraction of oxygen; HFNT: high flow nasal therapy; HR: heart rate; ICU: intensive care unit; PaCO_2_: partial pressure of carbon dioxide; PaO_2_: partial pressure of oxygen; RR: respiratory rate.

**Table 2S.** Main RCTs comparing different NRS techniques and interfaces in AHRF (chronological order of publication)

| **Author, year, N centers** | **N Patients** | **NRS techniques** | **Interface** | **Outcomes** |
| --- | --- | --- | --- | --- |
| Frat^14^, 2015, 23 ICUs | N=310, AHRF PaO_2_/FiO_2_ ≤300 and PaCO_2_ ≤ 45 | COT (N=94) vs. HFNT (N=106) vs. BiPAP (N=110) | Facemask | *EI rate at 28 days:* COT 47% vs. HFNT 38% vs. BiPAP 50% (p=0.18 for all the comparisons; OR 1.65, CI 95% 0.96-2.84 for BiPAP vs. HFNT);  *EI rate at 28 days (*PaO_2_/FiO_2_ ≤200)*:* COT 53 % vs. HFNT 35% vs. BiPAP 58% (p=0.009 for all the comparisons; OR 2.57, CI 95% 1.37-4.84 for BiPAP vs. HFNO); *ICU mortality:* COT 19% vs. HFNT 11% vs. BiPAP 25% (p=0.047 for all the comparisons; OR 2.55, CI 95% 1.21-5.35 for BiPAP vs. HFNT); 90 days mortality: COT 23% vs. HFNT 12% vs. BiPAP 28% (p=0.02 for all the comparisons; OR 2.50, CI 95% 1.31-4.78 for BiPAP vs. HFNT). |
| Patel^15^, 2016, 1 ICU | N=83, ARDS | CPAP or BiPAP | Facemask (N=39) vs. helmet (N=44) | *EI rate:* FM 61.5% vs. helmet 18.2% (p<0.001); *VFDs:* FM 12.5 vs. helmet 28 (p<0.001); *hospital mortality:* FM 48.7% vs. helmet 27.3% (p=0.04); *90 days mortality*: FM 56.4% vs. helmet 34.1% (p=0.02). |
| Nair, 2021^16^, 1 ICU | N=109, COVID-19 AHRF | HFNT (N=55) vs. BiPAP (N=54) | Facemask or helmet | *EI rate at 48h*: HFNT 20% vs. BiPAP 33% (p=0.12); *EI rate at 7 days*: HFNT 27% vs. BiPAP 46% (p=0.045); *hospital mortality:* HFNT 29% vs. BiPAP 46% (p=0.06). |
| Grieco, 2021^17^, 4 ICUs | N=109, COVID-19 AHRF | HFNT (N=55) vs. BiPAP (N=54) | Helmet | *Days free of respiratory support*: HFNT 18 [0-22] vs. BiPAP 20 [0-25] (p=0.26); *Days free of invasive ventilation*: HFNT 25 [4-28] vs. BiPAP 28 [13-28] (p=0.04); *EI rate:* HFNT 51% vs. BiPAP 30% (p=0.03); *hospital mortality:* HFNT 25% vs. BiPAP 24% (p>0.99). |
| Bouadma, 2022^18^, 19 ICUs | N=333, COVID-19 AHRF | COT (N=109) vs. HFNT (N=115) vs. CPAP (N=109) | Oronasal mask | *EI rate at 28 days:* COT 41.4% vs. HFNT 43.8% vs. CPAP 43% (p=0.85). |
| Arabi, 2022^19^, 6 ICUs | N=320, COVID-19 AHRF | BiPAP or HFNT or COT | Helmet (N=159) vs. usual respiratory support- Facemask, HFNT, COT- (N=161) | *EI rate at 28 days:* Helmet 27% vs. usual respiratory support 26% (p=0.85); *ICU* m*ortality:* Helmet 35% vs. usual respiratory support 37% (p=0.70); *H-*m*ortality:* Helmet 61% vs. usual respiratory support 64% (p=0.80). |
| Coudroy, 2022^20^, 29 ICUs | N=299, Immunocompromised patients with AHRF | HFNT (N=154) vs. BiPAP (N=145) | Facemask or helmet | *Mortality at 28 days:* HFNT 36% vs. BiPAP 35% (p=0.83); *discomfort after initiation (assessed by VAS):* greater with HFNT (p=0.04). |
| RENOVATE Investigators and the BRICNet Authors^13^, 2024, 33 centers (Eds, medical wards or ICUs) | N=485, AHRF subgroup | HFNT (N=249) vs. BiPAP (N=236) | Facemask | *Death or intubation (combined) within 7 days:* HFNT 32.5% vs. BiPAP 33.1% (OR 1.02, 95% CI 0.81-1.26; noninferiority posterior probability 0.999). |
| RENOVATE Investigators and the BRICNet Authors^13^, 2024, 33 centers (Eds, medical wards or ICUs) | N=50, Immunocompromised patients with AHRF subgroup | HFNT (N=28) vs. BiPAP (N=22) | Facemask | *Death or intubation (combined) within 7 days:* HFNT 57.1% vs. BiPAP 36.4% (OR 1.07, 95% CI 0.81-1.39; noninferiority posterior probability 0.989). |
| RENOVATE Investigators and the BRICNet Authors^13^, 2024, 33 centers (Eds, medical wards or ICUs) | N=882, COVID 19 AHRF subgroup | HFNT (N=435) vs. BiPAP (N=447) | Facemask | *Death or intubation (combined) within 7 days:* HFNT 51.3% vs. BiPAP 47.0% (OR 1.13, 95% CI 0.94-1.38; noninferiority posterior probability 0.997). |

List of abbreviations: AHRF: acute hypoxemic respiratory failure; BiPAP: bilevel positive airway pressure; CPAP: continuous positive airway pressure; COT: Conventional oxygen therapy; ED: emergency department; EI: endotracheal intubation; FiO_2_: inspired fraction of oxygen; HFNT: high flow nasal therapy; ICU: intensive care unit; NRS: non-invasive respiratory support; PaO_2_: partial pressure of oxygen; RR: respiratory rate.

**Table 3S.** Main RCTs comparing different non-invasive respiratory support techniques in AECOPD (chronological order of publication)

| **Author, year, N centers** | **N Patients** | **Techniques** | **Interface** | **Outcomes** |
| --- | --- | --- | --- | --- |
| Papachatzakis^21^, 2020, 1 ED | N=40, Hypercapnic respiratory failure | HFNT (N=20) vs. BiPAP (N=20) | Mask (unspecified if facemask or oronasal mask) | *Hospital LOS:* HFNT 11.5 vs. BiPAP 11 (p=0.655). |
| Cortegiani, 2020^22^, 9 centers (EDs, ICUs or Respiratory Units) | N=53, Hypercapnic respiratory failure | HFNT (N=24) vs. BiPAP (N=29) | Facemask or oronasal mask | *PaCO_2_ reduction at 2h:* HFNT -6.8 vs. BiPAP -9.5 (p=0.404); *PaCO_2_ reduction at 6h:* HFNT -9.5 vs. BiPAP -14.3 (p=0.096); *switch to* HFNT *or BiPAP:* HFNT 32.5% vs. BiPAP 7.7% (p=0.006). |
| Doshi, 2020^23^, 3 EDs | N=65, Hypercapnic respiratory failure | HFNT (N=34) vs. BiPAP (N=31) | Oronasal mask | *Change in pCO_2_ and pH over time*: ns difference; *treatment failure:* HFNT 23.5% vs. BiPAP 25.8% (p=1.000);  *EI rate:* HFNT 5.9% vs. BiPAP 16.1% (p=0.244) |
| RENOVATE Investigators and the BRICNet Authors, 2024^13^, 33 centers (Eds, medical wards or ICUs) | N=77, AECOPD with respiratory acidosis subgroup | HFNT (N=35) vs. BiPAP (N=42) | Facemask | *Death or intubation (combined) within 7 days:* HFNT 28.6% vs. BiPAP 26.2% (OR 1.05, 95% CI 0.79-1.36; noninferiority posterior probability 0.992). |
| Tan, 2024^24^, 2 ICUs | N=225, AECOPD with respiratory acidosis | HFNT (N=113) vs. BiPAP (N=112) | Oronasal mask | *Rate of treatment failure* (EI or a switch to the other study treatment modality): HFNT 25.7% vs. BiPAP 14.3% (p=0.033); *EI rate:* HFNT 14.2% vs BiPAP 5.4%, (p=0.026) |
| Luo, 2024^25^, 30 respiratory non-ICU wards | N=300, AECOPD with respiratory acidosis | Low intensity BiPAP (maximum IPAP 20 cmH_2_O to obtain Vt 6-10 mL/Kg) vs. high intensity BiPAP (maximum IPAP 30 cmH_2_O to obtain Vt 10-15 mL/Kg) | Oronasal mask (first choice), nasal mask (if  oronasal mask not tolerated) | *Criteria for the need of intubation*:* Low intensity BiPAP 13.7% vs. high intensity BiPAP 4.8 (p=0.004) |

List of abbreviations: BiPAP: bilevel positive airway pressure; CPAP: continuous positive airway pressure; COT: Conventional oxygen therapy; ED: emergency department; EI: endotracheal intubation; HFNT: high flow nasal therapy; ICU: intensive care unit; PaCO_2_: partial pressure of carbon dioxide; RR: respiratory rate.

*Criteria for the need of intubation were defined by (1) arterial pH < 7.25 with a PaCO2 level that increased by more than 20% compared with the baseline level or PaO_2_/FIO_2_ < 100 mm Hg; and (2) the presence of at least 1 of the following: clinical signs suggestive of severely decreased consciousness, use of accessory respiratory muscles or thoracoabdominal paradoxical movement, excessive respiratory secretions, aspiration or vomiting, bleeding in upper gastrointestinal tract, severe hemodynamic instability without response to fluid resuscitation and low-dose vasoactive agents, or ventricular or supraventricular arrhythmias; or (3) cardiac or respiratory arrest.

**Table 4S.** Main RCTs comparing different non-invasive respiratory support techniques in post-extubation prophylactic treatment (chronological order of publication)

| **Author, year, N centers** | **N Patients** | **Techniques** | **Interface** | **Outcomes** |
| --- | --- | --- | --- | --- |
| Hernández, 2016^26^, 3 ICUs | N=604, High risk for extubation failure* | HFNT (N=290) vs. BiPAP (N=314) | Facemask | *Reintubation at 72 h:* HFNT 22.8% vs. BiPAP 19.1% (difference between groups -3,7, CI -9 to ∞); *Postextubation respiratory failure at 72 h*: HFNT 26.9% vs. BiPAP 39.8% (difference between groups 12.9, CI 6.6 to ∞) |
| Thille, 2019^27^, 30 ICUs | N=641, High risk for extubation failure** | HFNT alone (N=302) vs. HFNT and BiPAP (N=339) | Facemask | *Reintubation at day 7:* HFNT alone 18.2% vs. HFNT and BiPAP 11.8% (p=0.020) |
| Hernández, 2022^28^, 2 ICUs | N=182, Very high risk for extubation failure*** | HFNT (N=90) vs. BiPAP (N=92) | Facemask | *Reintubation at day 7:* HFNT 38.9% vs. BiPAP 22.8% (p=0.019) |
| De Jong, 2023^29^, 39 ICUs | N=981, BMI ≥ 30 | HFNO or COT (N=491) vs. BiPAP (N=490) | Unspecified | *Treatment failure (switch to other treatment)*: HFNT/COT 26.5% vs. BiPAP 13.5% (p<0.001);  *Reintubation at 3 days*: HFNT/COT 12 % vs. BiPAP 10% (p 0.26); |
| Hernández, 2025, 2 ICUs | N=144, BMI ≥ 30 and intermediate risk for extubation failure**** | HFNT (N=72) vs. BiPAP (N=72) | Facemask | *Reintubation at day 7*: HFNT 33.3% vs. BiPAP NIV 23.6% (p = 0.27) |

List of abbreviations: BiPAP: bilevel positive airway pressure; BMI: body mass index; COT: Conventional oxygen therapy; HFNT: high flow nasal theraèhy; ICU: intensive care unit; PaCO_2_: partial pressure of carbon dioxide; RR: respiratory rate.

*High risk for extubation failure defined by the presence of at least 1 of the following risk factors: age >65 years; Acute Physiology and Chronic Health Evaluation II score higher than 12 points on extubation day; body mass index higher than 30; inadequate secretions management; difficult or prolonged weaning; more than 1 comorbidity; heart failure as primary indication for mechanical ventilation; moderate to severe chronic obstructive pulmonary disease; airway patency problems; or prolonged mechanical ventilation.

** High risk for extubation failure defined by the presence of at least 1 of the following risk factors: age >65 years; any underlying chronic cardiac; underlying chronic lung disease).

*** Very high risk for extubation failure defined by the presence of ≥ 4 of the following risk factors: age >65 years; heart failure as the primary indication for mechanical ventilation; moderate-to-severe chronic obstructive pulmonary disease; APACHE II score > 12 on extubation day; body mass index  > 30; airway patency problems; inadequate cough reflex or suctioning required > 2 times within 8 h before extubation; difficult or prolonged weaning (failing ≥ 1 attempt at disconnection from mechanical ventilation); ≥ 2 comorbidities; mechanical ventilation ≥ 7 days; hypercapnia (PaCO_2_ > 45 mmHg) at the end of the spontaneous breathing trial.

****Intermediate risk for extubation failure defined by the presence of **≤** 2 additional risk factors apart from obesity: age >65 years; heart failure as the primary indication for mechanical ventilation; moderate to severe chronic obstructive pulmonary disease; Acute Physiology and Chronic Health Evaluation II score >12 on extubation day; airway patency problems; inadequate cough reflex or suctioning required more than two times within 8 h before extubation; difficult or prolonged weaning; two or more comorbidities; length of mechanical ventilation ⩾7 days.

**REFERENCES**

1. Mehta S, Jay GD, Woolard RH, et al.: Randomized, prospective trial of bilevel versus continuous positive airway pressure in acute pulmonary edema. Critical Care Medicine 1997; 25:620

2. Bellone A, Monari A, Cortellaro F, Vettorello M, Arlati S, Coen D: Myocardial infarction rate in acute pulmonary edema: noninvasive pressure support ventilation versus continuous positive airway pressure. Crit Care Med 2004; 32:1860–5

3. Crane SD, Elliott MW, Gilligan P, Richards K, Gray AJ: Randomised controlled comparison of continuous positive airways pressure, bilevel non-invasive ventilation, and standard treatment in emergency department patients with acute cardiogenic pulmonary oedema. Emerg Med J 2004; 21:155–61

4. Park M, Sangean MC, Volpe MDS, et al.: Randomized, prospective trial of oxygen, continuous positive airway pressure, and bilevel positive airway pressure by face mask in acute cardiogenic pulmonary edema*: Critical Care Medicine 2004; 32:2407–15

5. Bellone A, Vettorello M, Monari A, Cortellaro F, Coen D: Noninvasive pressure support ventilation vs. continuous positive airway pressure in acute hypercapnic pulmonary edema. Intensive Care Med 2005; 31:807–11

6. Ferrari G, Olliveri F, De Filippi G, et al.: Noninvasive positive airway pressure and risk of myocardial infarction in acute cardiogenic pulmonary edema: continuous positive airway pressure vs noninvasive positive pressure ventilation. Chest 2007; 132:1804–9

7. Moritz F, Brousse B, Gellée B, et al.: Continuous positive airway pressure versus bilevel noninvasive ventilation in acute cardiogenic pulmonary edema: a randomized multicenter trial. Ann Emerg Med 2007; 50:666–75, 675.e1

8. Gray A, Goodacre S, Newby DE, et al.: Noninvasive ventilation in acute cardiogenic pulmonary edema. N Engl J Med 2008; 359:142–51

9. Ferrari G, Milan A, Groff P, et al.: Continuous positive airway pressure vs. pressure support ventilation in acute cardiogenic pulmonary edema: a randomized trial. J Emerg Med 2010; 39:676–84

10. Liesching T, Nelson DL, Cormier KL, et al.: fr. J Emerg Med 2014; 46:130–40

11. Osman A, Via G, Sallehuddin RM, et al.: Helmet continuous positive airway pressure vs. high flow nasal cannula oxygen in acute cardiogenic pulmonary oedema: a randomized controlled trial. Eur Heart J Acute Cardiovasc Care 2021; 10:1103–11

12. Marjanovic N, Piton M, Lamarre J, et al.: High-flow nasal cannula oxygen versus noninvasive ventilation for the management of acute cardiogenic pulmonary edema: a randomized controlled pilot study. Eur J Emerg Med 2024; 31:267–75

13. RENOVATE Investigators and the BRICNet Authors, Francio F, Weigert RM, et al.: High-Flow Nasal Oxygen vs Noninvasive Ventilation in Patients With Acute Respiratory Failure: The RENOVATE Randomized Clinical Trial. JAMA 2024 doi:10.1001/jama.2024.26244

14. Frat J-P, Thille AW, Mercat A, et al.: High-flow oxygen through nasal cannula in acute hypoxemic respiratory failure. N Engl J Med 2015; 372:2185–96

15. Patel BK, Wolfe KS, Pohlman AS, Hall JB, Kress JP: Effect of Noninvasive Ventilation Delivered by Helmet vs Face Mask on the Rate of Endotracheal Intubation in Patients With Acute Respiratory Distress Syndrome: A Randomized Clinical Trial. JAMA 2016; 315:2435–41

16. Nair PR, Haritha D, Behera S, et al.: Comparison of High-Flow Nasal Cannula and Noninvasive Ventilation in Acute Hypoxemic Respiratory Failure Due to Severe COVID-19 Pneumonia. Respir Care 2021; 66:1824–30

17. Grieco DL, Menga LS, Cesarano M, et al.: Effect of Helmet Noninvasive Ventilation vs High-Flow Nasal Oxygen on Days Free of Respiratory Support in Patients With COVID-19 and Moderate to Severe Hypoxemic Respiratory Failure: The HENIVOT Randomized Clinical Trial. JAMA 2021; 325:1731–43

18. Bouadma L, Mekontso-Dessap A, Burdet C, et al.: High-Dose Dexamethasone and Oxygen Support Strategies in Intensive Care Unit Patients With Severe COVID-19 Acute Hypoxemic Respiratory Failure: The COVIDICUS Randomized Clinical Trial. JAMA Intern Med 2022; 182:906–16

19. Arabi YM, Aldekhyl S, Al Qahtani S, et al.: Effect of Helmet Noninvasive Ventilation vs Usual Respiratory Support on Mortality Among Patients With Acute Hypoxemic Respiratory Failure Due to COVID-19: The HELMET-COVID Randomized Clinical Trial. JAMA 2022; 328:1063–72

20. Coudroy R, Frat J-P, Ehrmann S, et al.: High-flow nasal oxygen alone or alternating with non-invasive ventilation in critically ill immunocompromised patients with acute respiratory failure: a randomised controlled trial. Lancet Respir Med 2022; 10:641–9

21. Papachatzakis Y, Nikolaidis PT, Kontogiannis S, Trakada G: High-Flow Oxygen through Nasal Cannula vs. Non-Invasive Ventilation in Hypercapnic Respiratory Failure: A Randomized Clinical Trial. Int J Environ Res Public Health 2020; 17:5994

22. Cortegiani A, Longhini F, Madotto F, et al.: High flow nasal therapy versus noninvasive ventilation as initial ventilatory strategy in COPD exacerbation: a multicenter non-inferiority randomized trial. Crit Care 2020; 24:692

23. Doshi PB, Whittle JS, Dungan G, et al.: The ventilatory effect of high velocity nasal insufflation compared to non-invasive positive-pressure ventilation in the treatment of hypercapneic respiratory failure: A subgroup analysis. Heart Lung 2020; 49:610–5

24. Tan D, Wang B, Cao P, et al.: High flow nasal cannula oxygen therapy versus non-invasive ventilation for acute exacerbations of chronic obstructive pulmonary disease with acute-moderate hypercapnic respiratory failure: a randomized controlled non-inferiority trial. Crit Care 2024; 28:250

25. Luo Z, Li Y, Li W, et al.: Effect of High-Intensity vs Low-Intensity Noninvasive Positive Pressure Ventilation on the Need for Endotracheal Intubation in Patients With an Acute Exacerbation of Chronic Obstructive Pulmonary Disease: The HAPPEN Randomized Clinical Trial. JAMA 2024; 332:1709

26. Hernández G, Vaquero C, Colinas L, et al.: Effect of Postextubation High-Flow Nasal Cannula vs Noninvasive Ventilation on Reintubation and Postextubation Respiratory Failure in High-Risk Patients: A Randomized Clinical Trial. JAMA 2016; 316:1565

27. Thille AW, Muller G, Gacouin A, et al.: Effect of Postextubation High-Flow Nasal Oxygen With Noninvasive Ventilation vs High-Flow Nasal Oxygen Alone on Reintubation Among Patients at High Risk of Extubation Failure: A Randomized Clinical Trial. JAMA 2019; 322:1465

28. Hernández G, Paredes I, Moran F, et al.: Effect of postextubation noninvasive ventilation with active humidification vs high-flow nasal cannula on reintubation in patients at very high risk for extubation failure: a randomized trial. Intensive Care Med 2022; 48:1751–9

29. De Jong A, Bignon A, Stephan F, et al.: Effect of non-invasive ventilation after extubation in critically ill patients with obesity in France: a multicentre, unblinded, pragmatic randomised clinical trial. The Lancet Respiratory Medicine 2023; 11:530–9
